# Synthesis and Evaluation of a Monomethyl Auristatin E—Integrin α_v_β_6_ Binding Peptide–Drug Conjugate for Tumor Targeted Drug Delivery

**DOI:** 10.1021/acs.jmedchem.3c00631

**Published:** 2023-07-07

**Authors:** Ryan A. Davis, Tanushree Ganguly, Rebecca Harris, Sven H. Hausner, Luciana Kovacs, Julie L. Sutcliffe

**Affiliations:** † Department of Biomedical Engineering, University of California, Davis, One Shields Avenue, Davis, California 95616, United States; ‡ Department of Internal Medicine, Division of Hematology/Oncology, University of California, Davis, 4150 V Street, Sacramento, California 95817, United States; § Center for Molecular and Genomic Imaging, University of California, Davis, 451 Health Sciences Drive, Davis, California 95616, United States; ∥ Radiochemistry Research and Training Facility, University of California, Davis, 2921 Stockton Blvd., Suite 1760, Sacramento, California 95817, United States

## Abstract

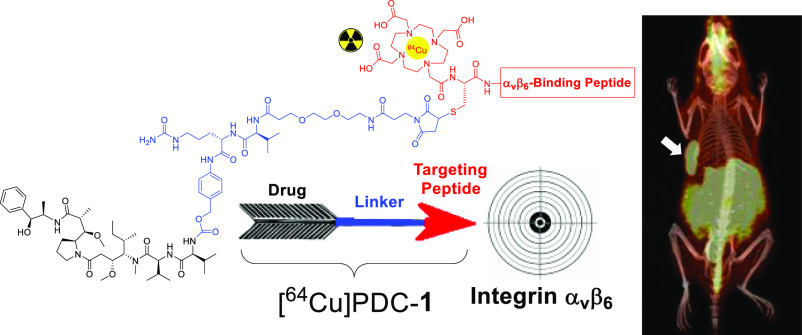

Many anticancer drugs exhibit high systemic off-target toxicities causing severe side effects. Peptide–drug conjugates (PDCs) that target tumor-specific receptors such as integrin α_v_β_6_ are emerging as powerful tools to overcome these challenges. The development of an integrin α_v_β_6_-selective PDC was achieved by combining the therapeutic efficacy of the cytotoxic drug monomethyl auristatin E with the selectivity of the α_v_β_6_-binding peptide (α_v_β_6_-BP) and with the ability of positron emission tomography (PET) imaging by copper-64. The [^64^Cu]PDC-**1** was produced efficiently and in high purity. The PDC exhibited high human serum stability, integrin α_v_β_6_-selective internalization, cell binding, and cytotoxicity. Integrin α_v_β_6_-selective tumor accumulation of the [^64^Cu]PDC-**1** was visualized with PET-imaging and corroborated by biodistribution, and [^64^Cu]PDC-**1** showed promising in vivo pharmacokinetics. The [^nat^Cu]PDC-**1** treatment resulted in prolonged survival of mice bearing α_v_β_6_ (+) tumors (median survival: 77 days, vs α_v_β_6_ (−) tumor group 49 days, and all other control groups 37 days).

## Introduction

Many of the current cancer treatment options are non-targeted and lack selectivity, affecting both the cancer and normal tissue.^1^ This uncontrolled killing of healthy cells results in high systemic off-target toxicity, severe side effects, and poor quality of life for patients.^1^ To overcome these challenges several tumor-targeting strategies have been explored including antibody–drug conjugates (ADCs) and peptide–drug conjugates (PDCs). Since 2019 only 9 ADCs have been FDA approved, including brentuximab vedotin, enfortumab vedotin, and polatuzumab vedotin, which are conjugated to monomethyl auristatin E (MMAE),^1−6^ while no PDC has yet gained regulatory approval.^7,8^ Although ADCs have demonstrated great promise, several challenges remain, notably the controlled site-specific chemical conjugation of the drug to the antibody, which often leads to ADC instability, poor antibody target affinity, and purification challenges.^2−4^ In addition, their large size can result in poor tumor penetration and long blood residence times, thereby further increasing systemic toxicity.^2−4^ To overcome some of these limitations, peptides have been investigated as delivery vehicles for the delivery of cytotoxic agents. Peptides are relatively easily synthesized by solid-phase peptide synthesis (SPPS), can be prepared in large quantities, and are readily modified to fine-tune affinity, selectivity, stability, and pharmacokinetics.^9^ The ease of modification makes them an ideal platform as a PDC, and their smaller size permits better tumor penetration and a shorter blood residence time which can reduce systemic toxicity.

Many tumor-specific cell surface receptors have been identified as therapeutic targets, among them the integrins which are a family of cell surface receptors that are involved in cell migration and invasion.^10,11^ Recently, the integrin α_v_β_6_ has garnered much attention as a target for both the detection as well as the treatment of cancers. The integrin α_v_β_6_ is an epithelial-specific cell surface receptor with low-to-no expression on healthy adult epithelium, but is highly overexpressed in many cancers, including some of the most lethal malignancies such as pancreatic cancer.^12−14^ Studies have shown that the integrin α_v_β_6_ plays a key role in carcinogenesis, where it is involved in cellular invasion, migration, angiogenesis, and adhesion to the extracellular matrix.^15^ Importantly, it has been identified as a prognostic indicator, with high expression level correlating to poor prognosis and overall survival for patients.^15^ Consequently, our group has developed and extensively studied the integrin α_v_β_6_-binding peptide (α_v_β_6_-BP), a peptide with nanomolar affinity and highly selective binding to integrin α_v_β_6_. The fluorine-18-labeled α_v_β_6_-BP was translated into the clinic to detect tumors in patients with breast, colon, lung, and pancreas cancer.^16^ The α_v_β_6_-BP rapidly binds to and is internalized into α_v_β_6_-expressing cells,^17−19^ and therefore, we now propose to use it as a chaperone for the selective delivery of the highly potent cytotoxic agent MMAE.

**Figure 1:**
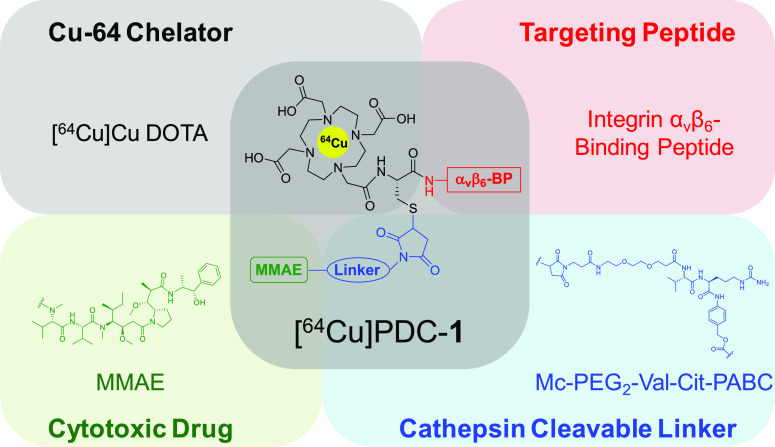
Structural components of the integrin α_v_β_6_ targeting [^64^Cu]PDC-**1**.

The design of the PDC (Figure 1) incorporates four key components: (1) the integrin α_v_β_6_ tumor-targeting peptide (α_v_β_6_-BP), (2) the cancer-specific cathepsin cleavable linker maleimide-PEG_2_-valine-citrulline-*para*-aminobenzylcarbamate (Mc-PEG_2_-Val-Cit-PABC),^20−23^ (3) the cytotoxic drug MMAE,^3,4^ and (4) a 2,2′,2″,2‴-(1,4,7,10-tetraazacyclododecane-1,4,7,10-tetrayl)tetraacetic acid (DOTA) chelator for copper-64 chelation. The PDC was evaluated for integrin α_v_β_6_-affinity by ELISA. It was radiolabeled with copper-64 to yield [^64^Cu]PDC-**1** which was tested for stability in mouse and human serum (1, 4, and 24 h; 37 °C), cell binding and internalization studies using the melanoma cell lines DX3puroβ6 (+) and DX3puro (−), and the pancreatic cell lines BxPC-3 (+) and MIA PaCa-2 (−). Cytotoxicity was tested by WST-1 assay, and apoptosis was correlated to caspase-3/7 activity. In vivo (PET/CT and biodistribution) of [^64^Cu]PDC-**1** was done in a paired DX3puroβ6/DX3puro as well as a BxPC-3 xenograft tumor mouse model. Therapeutic efficacy of [^nat^Cu]PDC-**1** was evaluated in mice bearing either DX3puroβ6 or DX3puro xenograft tumors.

## Results

### Chemistry & Radiochemistry

**Scheme 1:**
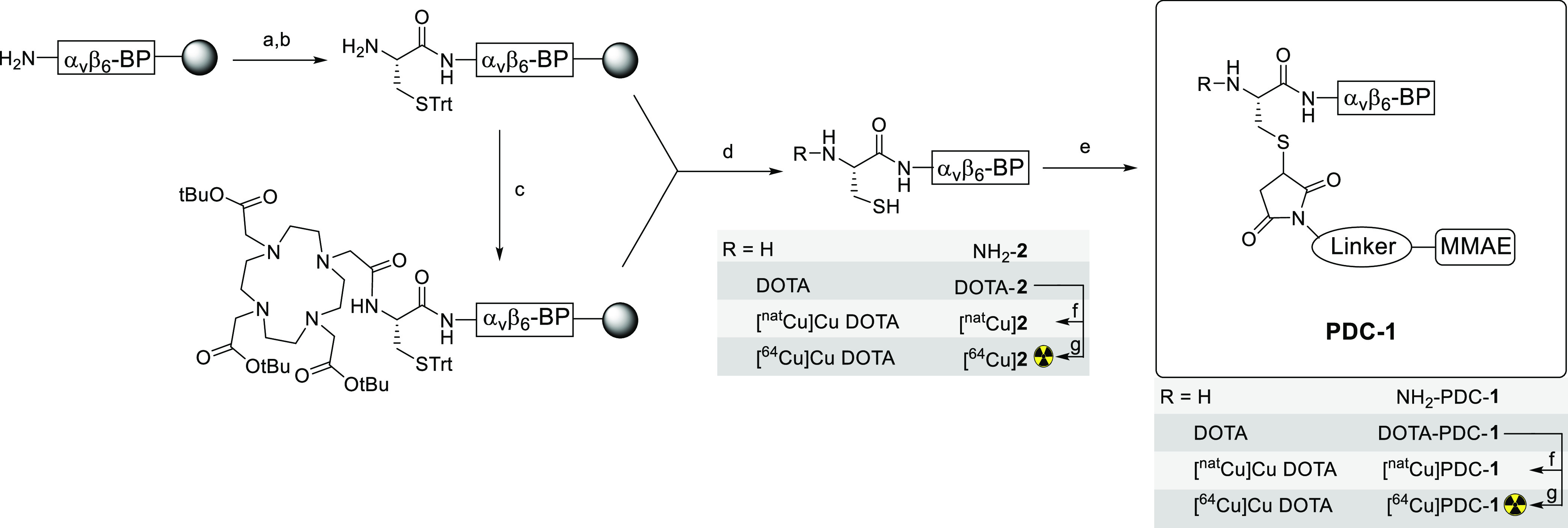
Synthetic Route for the PDC-**1** Synthesis scheme and reaction conditions: (a) Fmoc-Cys(Trt)-OH, HATU, DIPEA, DMF, (b) 20%-piperidine in DMF, (c) DOTA tris(*t*-butyl ester), HATU, DIPEA, DMF, (d) TFA, TIPS, H_2_O, (e) MMAE-Linker (Mc-PEG_2_-Val-Cit-PABC-MMAE), DMSO/pyridine (1/3), (f) CuSO_4_, H_2_O, (g) [^64^Cu]CuCl_2_, 1.0 M NH_4_OAc (pH = 8.0), 37 °C. α_v_β_6_-BP: PEG_28_-NAVPNLRGDLQVLAQRVART-PEG_28_

The α_v_β_6_-BP was modified to contain a cysteine for conjugation of the MMAE-maleimide linker (Scheme 1) and *N*-terminally capped with DOTA for radiolabeling with copper-64. Purified NH_2_-**2** peptide and DOTA-**2** were produced in 9 and 5% overall yield, respectively, from starting loading capacity of the resin. The conjugation of the MMAE-maleimide linker in solution was efficient and produced NH_2_-PDC-**1** and DOTA-PDC-**1** in 78 and 89% yield, respectively, from the respective purified, lyophilized peptide precursors (NH_2_-**2** and DOTA-**2**), in a 1:1 ratio of MMAE-per-peptide, in >99% purity after HPLC purification. The analytical data are: NH_2_-**2**, HPLC retention time (RT) = 17.13 min; matrix assisted laser desorption ionization time of flight (MALDI-TOF) *m*/*z*: calcd for C_214_H_404_N_37_O_86_S [M + H]^+^ 4901.8126; found, 4901.8124 (Figures S4 and S5); DOTA-**2**, HPLC RT = 17.17 min; MALDI-TOF *m*/*z*: calcd for C_230_H_430_N_41_O_93_S [M + H]^+^ 5287.9927; found, 5287.9900 (Figures S6 and S7); and [^nat^Cu]**2**, HPLC RT = 17.82 min; MALDI-TOF *m*/*z*: calcd for C_230_H_429_CuN_41_NaO_93_S [M + Na]^+^ 5373.9076; found, 5373.8769 (Figures S8 and S9). The analytical data of the PDCs are: NH_2_-PDC-**1**, HPLC RT = 19.13 min; MALDI-TOF *m*/*z*: calcd for C_286_H_516_N_49_O_104_S [M + H]^+^ 6337.5725; found, 6337.5759 (Figure S14 and S15); DOTA-PDC-**1**, HPLC RT = 19.02 min; MALDI-TOF *m*/*z*: calcd for C_302_H_541_N_53_NaO_111_S [M + Na]^+^ 6743.7998; found, 6743.7885 (Figure S16 and S17); and [^nat^Cu]PDC-**1**, HPLC RT = 19.58 min; MALDI-TOF *m*/*z*: calcd for C_302_H_541_CuN_53_O_111_S [M + H]^+^ 6783.7396; found, 6783.7199 (Figure S18 and S19). Radiolabeling with [^64^Cu]CuCl_2_ generated [^64^Cu]**2** and [^64^Cu]PDC-**1** in nearly quantitative yields (≥99%) in a molar activity of 18.5 GBq/μmol with high radiochemical purity of ≥98% (*n* = 1 and *n* = 6, respectively) (Figures S10–S13, S20–S23).

### Integrin α_v_β_6_ ELISA

The half-maximum inhibitory concentration (IC_50_) of DOTA-PDC-**1** against biotinylated latency associated peptide for integrin α_v_β_6_ was evaluated by competitive ELISA and determined to be IC_50_ = 18 ± 2 nM, demonstrating that the affinity was not affected by the DOTA-C-MMAE-linker modifications (IC_50_ [DOTA-α_v_β_6_-BP] = 28 ± 3 nM).^26^

### Serum stability

**Figure 2:**
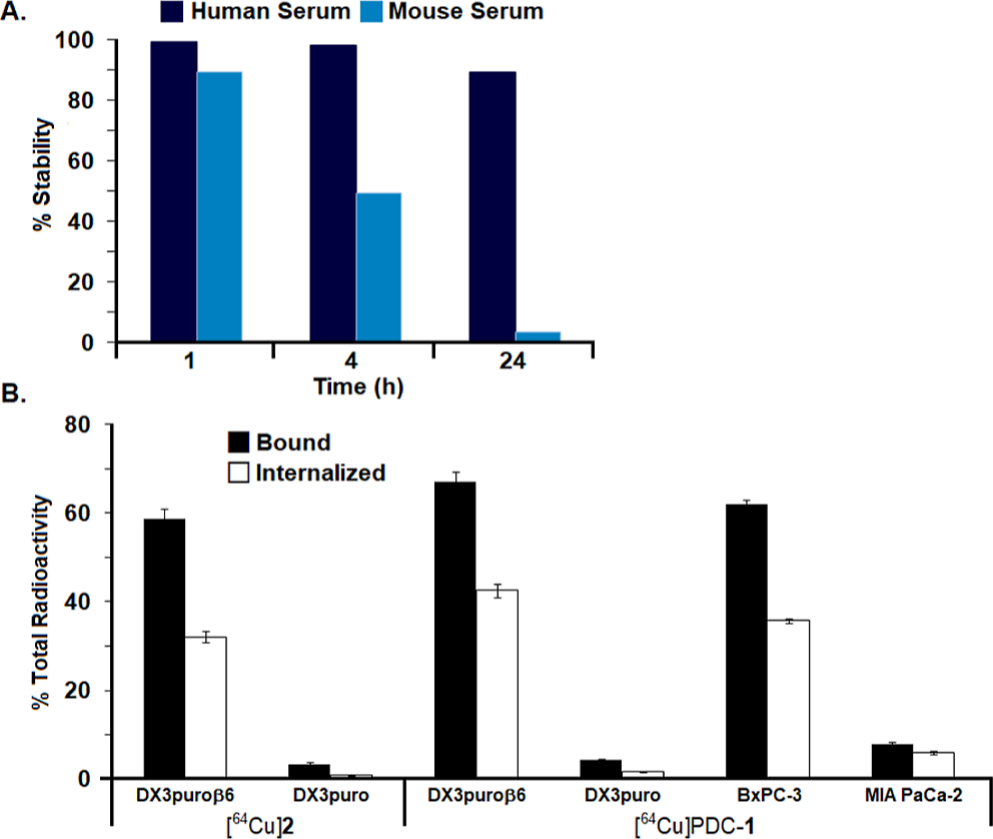
(A) Stability of [^64^Cu]PDC-**1** in human and mouse serum at 37 °C. (B) Cell binding and internalization of [^64^Cu]**2** and [^64^Cu]PDC-**1** in melanoma DX3puroβ6 (+) and DX3puro (−) cells and pancreatic BxPC-3 (+) and MIA PaCa-2 (−) cells.

The serum stability of [^64^Cu]PDC-**1** was measured in both human and mouse serum at 37 °C at 1, 4, and 24 h. [^64^Cu]PDC-**1** exhibited good stability in human serum (1 and 4 h >98%, 24 h 89%); degradation was more rapid in mouse serum (1 h 89%, 4 h 49%, 24 h 3%, Figure 2A).

### Cell Binding and Internalization Assay

Cell binding of [^64^Cu]PDC-**1** was high for the cell lines that exhibited high expression of integrin α_v_β_6_, with 67.0 ± 2.3% binding to the engineered melanoma DX3puroβ6 cells, and 62.0 ± 1.0% to pancreatic BxPC-3 cells (Figure 2B). Binding to cells with minimal to no expression of integrin α_v_β_6_ was low at 4.4 ± 0.1% to DX3puro cells and 7.9 ± 0.4% to pancreatic MIA PaCa-2 cells. Binding of [^64^Cu]PDC-**1** to DX3puroβ6 (+) and BxPC-3 (+) was reduced by adding increasing amounts of DOTA-PDC-**1**, illustrating that the α_v_β_6_-selective uptake could be blocked (Figure S3). Internalization of [^64^Cu]-PDC-**1** into cells was high with >50% of the bound radioactivity internalized for all cells expressing the integrin α_v_β_6_. In comparison, cell binding of [^64^Cu]**2** to DX3puroβ6 (+) cells was 58.8 ± 2.3, and 3.3 ± 0.4% to the DX3puro (−) cells.

### WST-1 Cell Viability Assay

**Figure 3:**
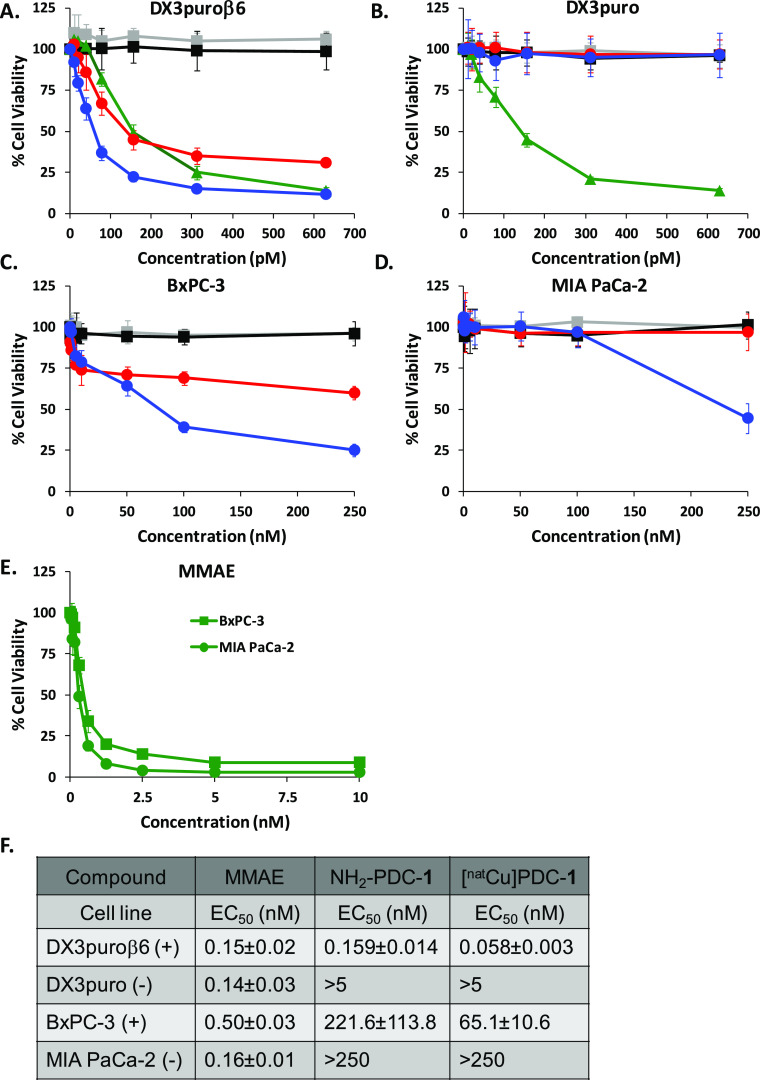
WST-1 cell viability assay. Peptides: NH_2_-**2** (gray ■) and [^nat^Cu]**2** (black ■); free, non-targeted MMAE (green ▲); PDCs: NH_2_-PDC-**1** (red ●) and [^nat^Cu]PDC-**1** (blue ●). **A**. DX3puroβ6 (+) and (B) DX3puro (−). (C). BxPC-3 (+), (D) MIA PaCa-2 (−), (E) MMAE in BxPC-3 and MIA PaCa-2. (F) Table of EC_50_ values for MMAE, NH_2_-PDC-**1**, and [^nat^Cu]PDC-**1**. Data are presented as the mean ± SD.

Both NH_2_-PDC-**1** and [^nat^Cu]PDC-**1** exhibited integrin α_v_β_6_-dependent cytotoxicity, only reducing cell viability of the α_v_β_6_-positive cells (Figure 3, red and blue, respectively). For [^nat^Cu]PDC-**1** high cytotoxicity was observed in DX3puroβ6 (+) cells (EC_50_: 0.058 ± 0.003 nM) with no observable cytotoxic effects in the DX3puro (−) cells, while free MMAE had almost equal cytotoxicity to both DX3puroβ6 (+) and DX3puro cells (−) (EC_50_: 0.14–0.15 nM, Figure 3A,B, green). The pancreatic cells also showed α_v_β_6_-dependent cytotoxicity for [^nat^Cu]PDC-**1** (EC_50_: BxPC-3 65.1 ± 10.6 nM, Figure 3C) and required high concentrations of ≥250 nM for noticeable cytotoxic effects in the minimally integrin α_v_β_6_-expressing MIA PaCa-2 cells (Figure 3D). Again, free, non-targeted MMAE exhibited nondiscriminatory cytotoxicity among the pancreatic cells with an effective concentration range of EC_50_ = 0.16–0.5 nM (Figure 3E). Peptides NH_2_-**2** and [^nat^Cu]**2** were not toxic to any cells (Figure 3, gray and black, respectively).

### Caspase-3/7 Activity Assay

**Figure 4:**
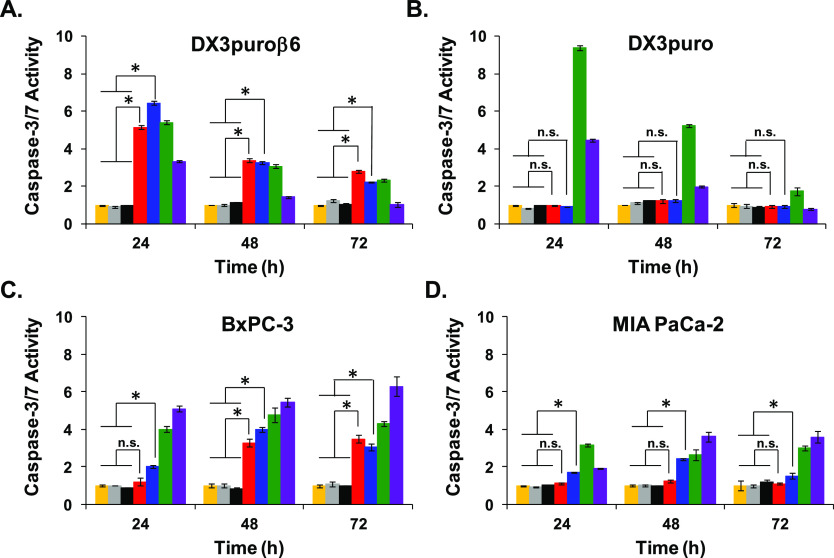
Caspase-3/7 activity determined by ApoTox-Glo Triplex Assay kit. Groups: untreated (yellow ■), NH_2_-**2** (gray ■), [^nat^Cu]**2** (black ■), NH_2_-PDC-**1** (red ■), [^nat^Cu]PDC-**1** (blue ■), MMAE (green ■), and positive control staurosporine (purple ■). Data are presented as the mean ± SD for (A) DX3puroβ6 (+), (B) DX3puro (−), (C) BxPC-3 (+), (D). MIA PaCa-2 (−). *Caspase-3/7 activity for treatment with NH_2_-PDC-**1** (red ■) or the [^nat^Cu]PDC-**1** (blue ■) are significantly different to untreated (yellow ■) and treatment with peptides NH_2_-**2** (gray ■) and [^nat^Cu]**2** (black ■), *P* < 0.05; n.s. = not significant.

The caspase-3/7 activity (Figure 4) is a measure of programmed cell death, and it was shown to correlate with the WST-1 cell viability assay (Figure 3). The treatment of cells with PDCs (NH_2_-PDC-**1**, [^nat^Cu]PDC-**1**) showed an α_v_β_6_-dependent increase in caspase-3/7 activity: for the DX3puroβ6 (+) and DX3puro (−) pair it resulted in a >5 times higher activity at 24 h for the DX3puroβ6 cells, with no observed change for the DX3puro cells (Figure 4A,B, 24 h red and blue, respectively). The increased caspase-3/7 activity was observed with the treatment of both NH_2_-PDC-**1** and [^nat^Cu]PDC-**1** at 24 h for the DX3puroβ6 cells and reached levels similar to non-targeted MMAE (Figure 4A, 24 h: green) and levels higher than that of the positive control staurosporine (Figure 4A, 24 h: purple). Conversely, DX3puro (−) cells, when treated with NH_2_-PDC-**1** or [^nat^Cu]PDC-**1**, produced caspase-3/7 activity levels indistinguishable from the untreated cells at all time points (Figure 4B, yellow); only MMAE (free, non-targeted) and the positive control staurosporine resulted in a large increase in caspase-3/7 activity in the DX3puro (−) cells (Figure 4B, 24 h: green and purple, respectively). The pancreatic BxPC-3 (+) cells also showed >3 fold increase in caspase-3/7 activity when treated with NH_2_-PDC-**1** or [^nat^Cu]PDC-**1** (Figure 4C, 48 h: red and blue, respectively), with levels approaching those of free MMAE (Figure 4C, 48 h: green). The pancreatic MIA PaCa-2 (−) cells showed little to no caspase-3/7 activity increase after treatment with NH_2_-PDC-**1** or [^nat^Cu]PDC-**1** (Figure 4D, red and blue, respectively), with levels remaining close to the untreated cells (Figure 4D, yellow). The staurosporine (purple) or free MMAE (green) provided increased caspase-3/7 activity in all cell lines regardless of integrin α_v_β_6_ expression, again showing the lack of integrin α_v_β_6_ selectivity for these non-targeted agents. The peptides containing no MMAE, i.e., NH_2_-**2** (gray) and [^nat^Cu]**2** (black), showed no effect on caspase-3/7 activity in all cell lines and were indistinguishable from untreated cells (yellow). Notably, a slight increase in caspase-3/7 activity was observed for the MIA PaCa-2 (−) cells when treated with the [^nat^Cu]PDC-**1** (Figure 4D, blue), which was not entirely unexpected since it had shown some initial toxic effect at the highest concentration by WST-1, however, the treatment with the NH_2_-PDC-**1** (red) resulted in no significant increase of caspase-3/7 activity at any time point (Figure 4D).

### PET Imaging and Biodistribution

**Figure 5:**
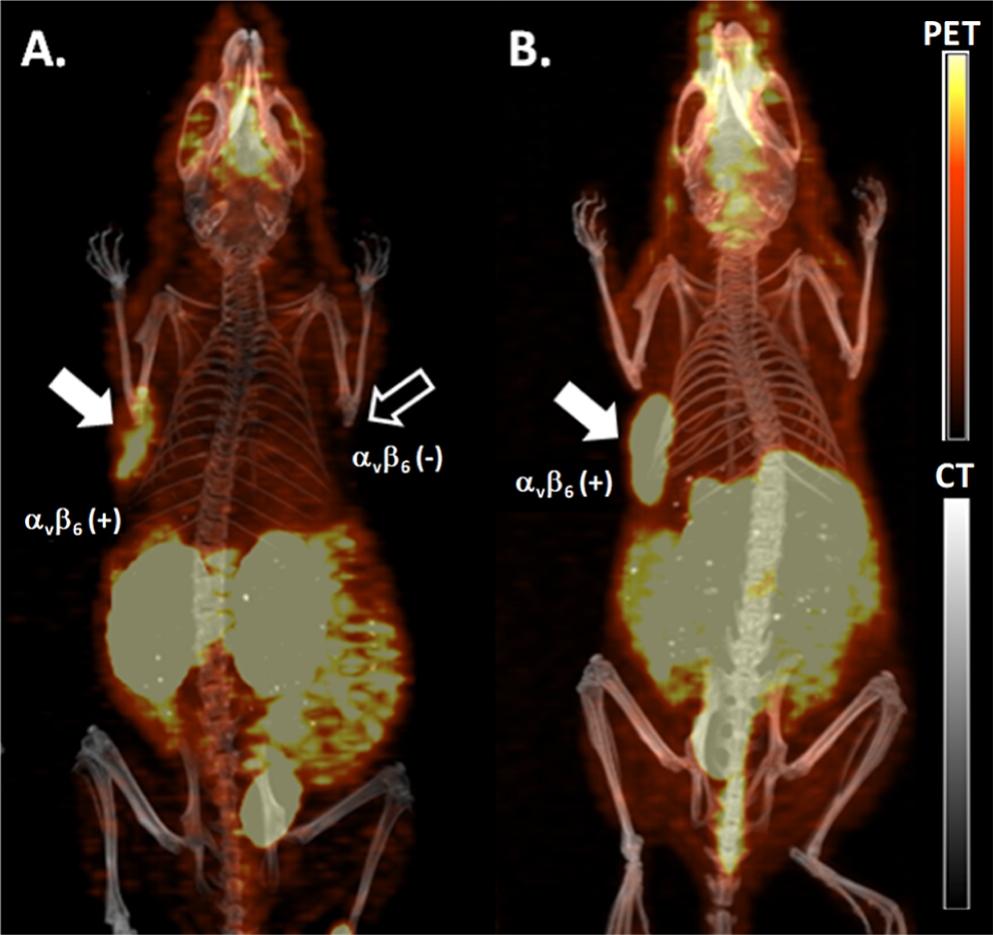
Maximum intensity projections (MIP) of PET/CT images obtained with [^64^Cu]PDC-**1** at 4 h p.i. (*n* = 4). (A) The paired DX3puroβ6/DX3puro xenograft tumor mouse model, showing selective uptake in α_v_β_6_ (+) tumor (filled arrow, DX3puroβ6). (B) The BxPC-3 pancreatic xenograft tumor mouse model, showing high tumor uptake. The PET data are shown in color scale and the CT data in gray.

**Figure 6:**
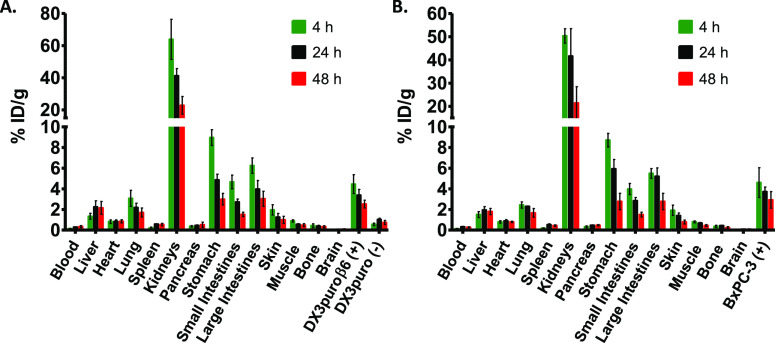
Biodistribution of [^64^Cu]PDC-**1**. (A) In the paired DX3puroβ6/DX3puro xenograft tumor mouse model (*n* = 4, 48 h: *n* = 9). (B) In the BxPC-3 pancreatic xenograft tumor mouse model (*n* = 4, 48 h: *n* = 6). Tissue uptake is expressed as the mean of the percentage of injected dose per gram of tissue ± SD.

[^64^Cu]PDC-**1** showed integrin α_v_β_6_-dependent targeting and accumulation with clear visualization of both the DX3puroβ6 (+) and BxPC-3 (+) tumors by positron emission tomography (PET) imaging, along with no observable uptake in the DX3puro (−) tumor (Figure 5). The PET images further showed high uptake in the kidneys, and some uptake in the gastrointestinal tract (stomach, small and large intestines, Figure 5). The biodistribution of [^64^Cu]PDC-**1** confirmed the α_v_β_6_-selective tumor accumulation, with 4.46 ± 0.91% ID/g in the DX3puroβ6 (+) tumor at 4 h vs 0.56 ± 0.12% ID/g in the DX3puro (−) tumor (ratio = 8:1; Figure 6A, Table S3). The BxPC-3 (+) tumor also exhibited a similarly high accumulation (4.61 ± 1.44% ID/g at 4 h; Figure 6B and Table S4). Moderate tumor washout was observed at later time points for both tumor models; it did reach significance at 48 h for the DX3puroβ6 tumor (4.46 ± 0.91% ID/g at 4 h to 3.39 ± 0.56% and 2.53 ± 0.37% ID/g at 24 h and 48 h, respectively, 4 to 48 h: *P* = 0.0002). For the BxPC-3 tumor, the uptake went from 4.61 ± 1.44% ID/g at 4 h to 3.73 ± 0.44 and 2.93 ± 0.80% ID/g, at 24 and 48 h, respectively (4 to 48 h: *P* = 0.054; Figure 6). Uptake of [^64^Cu]PDC-**1** was successfully blocked by pre-administration of DOTA-**2** (205 nmol) 10 min prior to administration of [^64^Cu]PDC-**1**, resulting in 87–91% reduced uptake in the α_v_β_6_ (+) tumors down to the level of the DX3puro (−) tumor (0.42 ± 0.04% ID/g; vs DX3puroβ6: 0.39 ± 0.04% ID/g and BxPC-3: 0.61 ± 0.05% ID/g at 4 h post injection; p.i.), thus demonstrating integrin α_v_β_6_-selective targeting in vivo (Table S6, Figure S24 ). Clearance from the blood was rapid, resulting in α_v_β_6_ (+) tumor/blood ratios of ≥32:1 at 4 h (Table S5). [^64^Cu]PDC-**1** primarily cleared through the kidneys, from 50 to 64% ID/g at 4 h to ≤25% ID/g at 48 h (Figure 6). Some uptake was observed in the gastrointestinal tract (Figure 6), with the stomach dropping from 9% ID/g at 4 h to ≤3% ID/g at 48 h, the large intestines from 6% ID/g at 4 h to 3% ID/g at 48 h, and the small intestines from 4 to 5% ID/g at 4 h to 1.5 %ID/g at 48 h with elimination in the fecal matter (12% ID/g at 4 h to 1.5% ID/g at 48 h). Accumulation in the liver remained steady between 1.5 and 2.2% ID/g at 4 to 48 h, and uptake in other organs such as muscle (≤0.9% ID/g) and pancreas (≤0.5% ID/g) was low at all time points (Figure 6).

### Therapy Study

**Figure 7:**
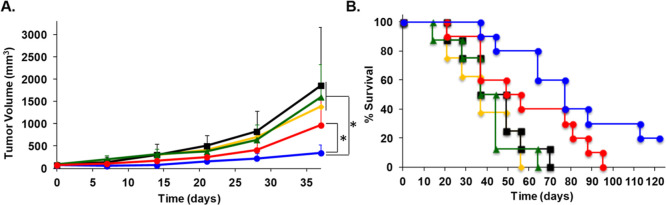
(A) Average tumor volume over time. (B) Kaplan–Meier survival plot. Treatment groups: saline (yellow ◆); [^nat^Cu]**2** ( black ■); and MMAE (green ▲), *n* = 8/group (each group consisting of half DX3puroβ6 (+) and half DX3puro (−) tumors). Treatment groups with [^nat^Cu]PDC-**1**: DX3puroβ6 (+) tumors (blue ●) and DX3puro (−) tumors (red ●), *n* = 10/group. *Average tumor volumes of all groups relative to the [^nat^Cu]PDC-**1** treated DX3puroβ6 tumor bearing mice (blue ●) are significantly different, (*P* < 0.05, day 37).

Mice treated with [^nat^Cu]PDC-**1** had slower tumor growth compared to the groups receiving saline, non-drug bearing peptide [^nat^Cu]**2**, or free, non-targeted MMAE (Figure 7). At 37 days post treatment, all DX3puroβ6 (+) tumor-bearing mice treated with [^nat^Cu]PDC-**1** were alive and had significantly lower mean tumor volumes compared to the control groups (saline vs [^nat^Cu]PDC-**1**, *P* < 0.0001; [^nat^Cu]**2** vs [^nat^Cu]PDC-**1**, *P* = 0.0001; MMAE vs [^nat^Cu]PDC-**1**, *P* = 0.0026; Figure 7A). The mean tumor volume at day 37 for the DX3puroβ6 (+) bearing mice treated with [^nat^Cu]PDC-**1** was significantly >2.75 times smaller than the equally treated DX3puro (−) tumors (*P* = 0.0099; Figure 7A); at the same time point the [^nat^Cu]PDC-**1**-treated DX3puroβ6 (+) mean tumor volume was >4 times smaller than all other treatment groups (saline, [^nat^Cu]**2**, MMAE). All mice in the groups treated with saline, non-drug bearing peptide [^nat^Cu]**2**, or free, non-targeted MMAE had met an end point criterion (≥2 cm in any direction and/or tumor ulceration) by 56 days, 70 days, and 64 days from start of treatment, respectively, with all these groups having the same median survival of 37 days (Figure 7B). The DX3puro (−) tumor bearing mice treated with [^nat^Cu]PDC-**1** had a median survival of 49 days, with all mice reaching an end point at 95 days, while those bearing DX3puroβ6 (+) tumors treated with [^nat^Cu]PDC-**1** had a median survival of 77 days, and a 20% survival at the end of the study (day 122, Figure 7B). No significant differences of the average body weight were observed between any of the groups, indicating no significant adverse events or high systemic toxicity from the [^nat^Cu]PDC-**1** (Figure S25).

## Discussion

Most standard chemotherapies do not distinguish cancerous cells from healthy cells, leading to less than ideal therapeutic efficacy and high systemic off-target toxicity. Tumor-targeted drug delivery approaches, such as PDCs, can improve accumulation of the therapeutic in the diseased tissue, reduce damage to healthy tissues and minimize unwanted side-effects. PDCs have been developed for targeting a wide range of receptors, including integrins,^27−42^ with a variety of cytotoxic agents including doxorubicin (Dox), paclitaxel (PXT), camptothecin (CPT), and MMAE.^1,7,27,43−45^ One emerging therapeutic target in oncology is the integrin α_v_β_6_, a cell surface receptor highly overexpressed in a wide range of malignancies with little to no expression on normal tissue.^13−16^ The integrin α_v_β_6_ is present in approximately 90% of pancreatic cancers and nearly all cases of metastatic disease.^13−16^ Pancreatic cancer remains one of the most lethal malignancies worldwide with a 5 year survival of less than 10%,^46^ in part due to limited treatment options. Surgery is the only cure and unfortunately less than 20% of patients are eligible for resection at the time of diagnosis due to the presence of metastasis.^13−16^ A clear unmet need for more effective and targeted treatments exists. We previously demonstrated that the α_v_β_6_-BP identified both primary and metastatic disease in a range of carcinomas.^16^ These data suggest that the development of an integrin α_v_β_6_-targeted PDC based on the α_v_β_6_-BP for selective delivery of highly cytotoxic agents like MMAE holds great promise.

MMAE inhibits tubulin assembly with cytotoxic activity in the picomolar range and is extremely lipophilic, preventing its use as a therapy due to high systemic toxicity.^3,4,47^ Efforts to overcome these high systemic toxicities include linking peptides to MMAE via protease-cleavable linkers. The linker choice is important because it governs the successful release of the cytotoxic agent. If the linker is too stable, release of the cytotoxic agent will be hindered, providing poor efficacy,^3,48^ and if the linker has low stability, non-specific release of the cytotoxic agent will occur, leading to increased systemic off-target toxicities and ineffective treatment.^3,48^ We chose the Mc-Val-Cit-PABC cleavable linker because it combines high stability in human plasma^49^ with rapid hydrolysis by lysosomal enzymes such as cathepsin B, an enzyme that is upregulated in cancer cells,^20−23^ resulting in the release of MMAE in its unaltered form.^21^ Standard SPPS combined with a site-specific Michael addition enabled the robust synthesis of the α_v_β_6_-BP-linker-MMAE-conjugate (PDC-**1**), and radiolabeling with copper-64 yielded [^64^Cu]PDC-**1** which enabled the quantitative assessment of cell binding, internalization, and in vivo pharmacokinetics.

[^64^Cu]PDC-**1** demonstrated integrin α_v_β_6_ receptor selective binding and internalization in vitro. [^nat^Cu]PDC-**1** also demonstrated integrin α_v_β_6_ selective cytotoxicity; for example, the DX3puroβ6 cells, having the highest integrin α_v_β_6_ expression, had an EC_50_ = 0.058 ± 0.003 nM, the intermediate integrin α_v_β_6_-expressing BxPC-3 had an EC_50_ = 65.1 ± 10.6 nM, the low expressing MIA PaCa-2 cells showed low cytotoxicity (EC_50_ > 250 nM) and the non-expressing DX3puro cells exhibited no observable cytotoxic effects. In contrast, the free, non-targeted MMAE was highly cytotoxic to all cells, having an EC_50_ of 0.14–0.5 nM. The in vitro efficacy of [^nat^Cu]PDC-**1** was comparable to the integrin α_v_β_6_-targeting PDC containing the cytotoxic drug tesirine (PDC, SG3299) that was previously reported to have an EC_50_ = 4.19–5.37 nM in α_v_β_6_-expressing cells, including in the engineered melanoma cell line A375Pβ6 and the pancreatic Capan-1 (EC_50_ = 4.19 ± 3.76 and 5.37 ± 5.23 nM, respectively).^42^ The tesirine-PDC (SG3299), when compared to the non-targeting scrambled PDC, tesirine-PDC (SG3511), provided a 15:1 ratio for selective cytotoxicity toward A375Pβ6 (+) melanoma cells, but the targeting tesirine-PDC (SG3299) also had relatively high cytotoxicity to α_v_β_6_-null engineered melanoma cells A375Ppuro and Panc-1 cells (EC_50_ = 30.6 ± 18.8 nM and 175.6 ± 115.7 nM, respectively).^42^ By comparison, in the present study, NH_2_-PDC-**1** was >31-fold and [^nat^Cu]PDC-**1** was >86-fold more cytotoxic toward the melanoma DX3puroβ6 (+) than the DX3puro (−) cells. Other integrin α_v_β_3_ and α_v_β_5_ targeting camptothecin (CPT) PDCs have shown less favorable in vitro efficacy of EC_50_ = 0.16–27 μM,^34^ with some integrin α_v_β_3_ targeting α-amanitin-PDCs exhibiting non-selective cytotoxicity.^41^ Piarulli et al. showed that MMAE-PDCs targeting integrin α_v_β_3_ produced cytotoxicities with EC_50_ = 11–400 nM, concluding they had a promising candidate for in vivo experiments to obtain evidence of accumulation at the tumor site.^40^

Indeed, few studies show biodistribution data for the PDCs, with limited examples including tritium or iodine-125 radiolabeled PDCs; however, these have limitations for noninvasive imaging and tracking.^29,50^ By contrast, radiolabeling the PDC with copper-64 enabled us to noninvasively image the [^64^Cu]PDC-**1** with PET, which demonstrated integrin α_v_β_6_-selective uptake in tumors that was corroborated by biodistribution studies (% ID/g, 4 h: DX3puroβ6 (+) 4.46 ± 0.91; BxPC-3 (+) 4.61 ± 1.44; DX3puro (−) 0.56 ± 0.12). Wang et al. described a similar radiolabeling approach with copper-64 to image integrin α_v_β_3_-targeted delivery of a bicyclic-RGD peptide, CDCRGDCFC (RGD4C), linked to the protein tumor necrosis factor (TNF) as the therapeutic agent.^32^ They demonstrated TNF-PDC accumulation in an MDA-MB-435 breast cancer xenograft tumor model (3.94 ± 0.48% ID/g at 4 h), and approximately double that uptake in a higher α_v_β_3_-expressing glioblastoma U87MG xenograft mouse model (8.11 ± 0.88% ID/g at 4 h); however, high liver accumulation of 16.22 ± 1.46% ID/g at 20 h was also observed.^32^ For [^64^Cu]PDC-**1**, minimal liver accumulation was observed (1.5–2.2% ID/g between 4 and 48 h, p.i.). Although the preliminary results are promising, the [^64^Cu]PDC-**1** pharmacokinetic profile still has its limitations of fast clearance, moderate tumor accumulation with some washout, and some off-target uptake in the gastrointestinal tract and high kidney accumulation 41.6% ID/g at 24 h).

Building on the encouraging in vitro and in vivo data suggesting selective integrin α_v_β_6_ targeting, the PDC-**1** was further evaluated for therapeutic efficacy. To permit a direct side-by-side comparison, this was done with DX3puroβ6 (+) or DX3puro (−) tumor bearing mice. Treatment with [^nat^Cu]PDC-**1** suppressed DX3puroβ6 (+) tumor growth and prolonged median survival to 77 days, compared to 49 days for the DX3puro (−) tumor-bearing mice, and >2-fold longer than other treatment groups (saline, non-drug bearing peptide [^nat^Cu]**2**, or free, non-targeted MMAE: median survival 37 days). The [^nat^Cu]PDC-**1** treated DX3puroβ6 tumor cohort had 20% remaining alive at the end of the study (122 days). Notably, the [^nat^Cu]PDC-**1** treatment did not cause adverse systemic side-effects when administered four times at 6 mg/kg (0.9 μmol/kg), as the mice maintained healthy body weight during the course of the study. This concentration corresponds to 0.64 mg/kg of free MMAE, i.e., close to the LD_50_ for free MMAE of 1 mg/kg (1.4 μmol/kg),^51^ thus highlighting the successful administration of a highly cytotoxic agent safely as part of a targeted PDC at concentrations that would be systemically toxic when administered alone.

## Conclusion

We developed the [^64^Cu]PDC-**1** by combining the highly cytotoxic drug MMAE with the highly selective integrin α_v_β_6_-BP, with the goal to reduce off-target toxicity of the drug whilst retaining therapeutic efficacy. In vitro testing demonstrated integrin α_v_β_6_-dependent binding, internalization, and cytotoxicity with high stability in human serum at 37 °C. PET/CT imaging of [^64^Cu]PDC-**1** showed integrin α_v_β_6_-selective tumor accumulation and visualization, and the biodistribution confirmed a favorable pharmacokinetic profile with rapid blood clearance and renal excretion. In vivo therapeutic efficacy studies displayed >2-fold improved overall survival of mice bearing DX3puroβ6 (α_v_β_6_ +) tumors compared to the control groups. Different dosing regimens are currently under evaluation with the goal to develop a highly effective, integrin α_v_β_6_-targeted PDC therapeutic for a wide range of carcinomas.

## Experimental Section

Reagent lists and commercial sources along with additional method details are described in the Supporting Information (S4–S36).

### Analytical Methodology

Characterization of purity and stability were confirmed using an analytical C_12_-reverse-phase (RP) high-pressure liquid chromatography (HPLC) column (Jupiter Proteo, 250 mm × 4.6 mm × 4 μm; Phenomenex, Torrance, CA) at a 1.5 mL/min flow rate. All reverse phase high performance liquid chromatography (RP-HPLC) was carried out on a Beckman Coulter Gold HPLC equipped with a 2 mL injection loop. RP-HPLC was monitored by UV detector at a wavelength of 220 nm; a serially connected γ-detector was used to monitor radioactivity. The mobile phase was a gradient starting at 9% acetonitrile in water containing 0.05% trifluoroacetic acid (TFA; EMD, Merck Millipore, Burlington, MA) held for 2 min, followed by linear ramp up to 81% acetonitrile over 30 min (for a total run time of 32 min till reaching 81%, Table S1). Purification of peptides was done by semi-preparative RP HPLC (C_12_: Jupiter Proteo column, 250 mm × 10 mm × 10 μm, Phenomenex) at a flow rate of 3 mL/min using the same gradient solvent system. After HPLC purification all peptides were confirmed by analytical HPLC to be >95% pure, and identity was confirmed by mass spectrometry at the UC Davis Mass Spectrometry Facility using a MALDI-TOF spectrometer (UltraFlextreme; Bruker, Billerica, MA) in positive ionization mode with a sinapic acid matrix (Sigma-Aldrich).

### Chemical Synthesis

The α_v_β_6_-BP (NH_2_-PEG_28_-NAVPNLRGDLQVLAQRVART-PEG_28_) was built by SPPS on NovaSyn TGR resin as previously described^16^ (Scheme 1). Following the α_v_β_6_-BP synthesis, the *N*-terminal Fmoc was removed and a reactive handle introduced by reacting resin-bound α_v_β_6_-BP (100 mg, 0.0088 mmol) with Fmoc-Cys(Trt)-OH (35 mg, 0.06 mmol), HATU (20 mg, 0.053 mmol) and DIPEA (25 μL, 0.14 mmol) in DMF (1 mL), followed by Fmoc removal. Next, the peptidyl resin was divided; one portion provided the peptide NH_2_-C-α_v_β_6_-BP (NH_2_-**2**), and the other afforded DOTA-NH-C-α_v_β_6_-BP (DOTA-**2**) after DOTA tris(*t*-butyl ester) conjugation (20 mg, 0.035 mmol) with HATU (10 mg, 0.026 mmol) and DIPEA (10 μL, 0.057 mmol) in DMF. The peptides (NH_2_-**2** and DOTA-**2**) were deprotected and removed from the resin using a mixture of trifluoroacetic acid (TFA), triisopropylsilane (TIPS), and water (TFA/TIPS/water, v/v/v, 95/2.5/2.5) for 3 h. Once cleaved, both peptides were purified using the semi-preparative RP-HPLC. Purified peptides were then conjugated to the MMAE-linker (Mc-PEG_2_-Val-Cit-PABC-MMAE) via Michael addition between the cysteine sulfhydryl and the maleimide in dimethylsulfoxide and pyridine (DMSO/pyr, 1/3, v/v) for 4 h as follows: the NH_2_-**2** (6.5 mg, 0.0013 mmol) was reacted with MMAE-linker (2.5 mg, 0.0017 mmol) in DMSO/pyr (1 mL) and DOTA-**2** (27 mg, 0.0051 mmol) with MMAE-linker (10 mg, 0.0069 mmol) in DMSO/pyr (2 mL). Crude reaction solutions were diluted with water (5 and 10 mL, respectively) and lyophilized. The lyophilized oils were then purified by RP-HPLC to afford NH_2_-PDC-**1** and DOTA-PDC-**1** in 78 and 89% yield, respectively, from the starting purified lyophilized peptide. The non-radioactive natural copper compounds were generated by reacting DOTA-PDC-**1** or DOTA-**2** with excess copper sulfate (CuSO_4_) in water, followed by RP-HPLC purification and confirmation by MALDI-TOF.

### Radiochemical Synthesis

DOTA-**2** (5 μg, 0.0009 μmol) was dissolved in metal free water (10 μL) and added to a solution of [^64^Cu]CuCl_2_ (0.0167 GBq) in 1.0 M ammonium acetate buffer (NH_4_OAc, 50 μL, pH = 8.0), and reacted at 37 °C for 30 min. DOTA-PDC-**1** (120 μg, 0.018 μmol) was dissolved in metal free water (120 μL) and added to a solution of [^64^Cu]CuCl_2_ (0.333 GBq) in 1.0 M ammonium acetate buffer (NH_4_OAc, 55 μL, pH = 8.0), and reacted at 37 °C for 30 min at a molar activity of 18.5 GBq/μmol. For analysis, an aliquot of the reaction mixture (≤1 μL; 0.25 MBq) was quenched with 0.1 M ethylenediaminetetraacetic acid (EDTA, 50 μL), radiochemical purity analyzed by analytical RP-HPLC, and identity confirmed by co-injection with non-radioactive [^nat^Cu]**2** or [^nat^Cu]PDC-**1**, respectively. Both [^64^Cu]**2** and [^64^Cu]PDC-**1** were obtained in ≥98% radiochemical purity and used for formulation without further purification.

### Serum Stability

Mouse serum or human serum (0.5 mL) was combined with an aliquot of [^64^Cu]PDC-**1** (≤25 μL, 3.9–4.7 MBq) and incubated at 37 °C. At each time point (1, 4, and 24 h), an aliquot (50–200 μL) was taken, proteins precipitated with absolute ethanol and removed by centrifugation at 1500*g* for 4 min. The ethanol solution was diluted with water (1 mL) and analyzed by RP-HPLC.

### WST-1 Cell Viability Assay

Cell viability was measured after treatment with either NH_2_-**2**, [^nat^Cu]**2**, MMAE (free, non-targeted drug), NH_2_-PDC-**1**, or [^nat^Cu]PDC-**1** in DX3puroβ6 (+) and DX3puro (−) cells at variable concentrations up to 5 nM, and in BxPC-3 (+) and MIA PaCa-2 (−) cells at variable concentrations up to 250 nM, or with MMAE at concentrations up to 10 nM. Cells were seeded in a 96 well plate at a density of 6000 cells/well for DX3puroβ6 and DX3puro, and at a density of 10,000 cells/well for BxPC-3 and MIA PaCa-2. DMEM media was used for all cells except for BxPC-3 (RPMI 1640 media). Cells (*n* = 6–8 wells/cell type/compound) were treated with different concentrations of NH_2_-**2**, [^nat^Cu]**2**, MMAE, NH_2_-PDC-**1**, or [^nat^Cu]PDC-**1** dissolved in the respective media, as well as their respective media (no treatment) for 48 h (37 °C, 5% CO_2_), after which the media was removed; cells were washed twice with media (200 μL) and re-incubated in media (37 °C, 5% CO_2_) for 24 h. The media was then removed and the WST-1 reagent was added to each well, and the cells were incubated for 2 h at 37 °C. The 96 well plates were read at 450 nm by a Multiscan Ascent microplate reader. The percent cell viability was normalized to untreated cells (set as 100% viability) for each cell line.

### Caspase-3/7 Activity Assay

Caspase-3/7 activity was analyzed using an ApoTox-Glo Triplex Assay kit. Cells were seeded in a 96 well plate at the same density and using the same respective media as described for the WST-1 assay and incubated overnight (37 °C, 5% CO_2_). DX3puroβ6 and DX3puro cells were treated with 1 nM of MMAE (free, non-targeted drug) or 0.625 nM of the other compounds: NH_2_-**2**, [^nat^Cu]**2**, NH_2_-PDC-**1**, or [^nat^Cu]PDC-**1**. BxPC-3 and MIA PaCa-2 cells were treated with 10 nM of MMAE or 250 nM of the other compounds: NH_2_-**2**, [^nat^Cu]**2**, NH_2_-PDC-**1**, or [^nat^Cu]PDC-**1**. All cells were treated with 100 nM of staurosporine as a positive control.^24^ Untreated cells (media) were used as a measure of endogenous caspase-3/7 activity (normalized to 1). Cells were treated (*n* = 4/cell line/compound/time) for 24, 48, or 72 h (37 °C, 5% CO_2_) prior to washing. After treatment, the media was removed, cells were washed, and Caspase-Glo 3/7 reagent was added, and incubated for 1 h at room temperature. Caspase-3/7 activity was analyzed by measuring luminescence with a Fluoroskan FL microplate reader according to the manufacturer’s protocol.

### In Vivo Studies

All animal procedures conformed to the Animal Welfare Act and were approved by the University of California Davis Institutional Animal Care and Use Committee. All mice used for in vivo work were female athymic nude mice (6–8 weeks old) purchased from Charles River Laboratories (Wilmington, MA). For PET imaging and biodistribution studies, female athymic nude mice (6–8 weeks old) were injected subcutaneously with 3 × 10^6^ DX3puro and 3 × 10^6^ DX3puroβ6 cells in serum free DMEM on the right and left flank, respectively, or with 5 × 10^6^ BxPC-3 cells in serum free RPMI 1640/Matrigel (1/1 v/v). Studies commenced once tumors reached a maximum diameter of ∼0.5 cm, approximately 3 weeks after inoculation. Food and water were available ad libitum. [^64^Cu]PDC-**1** was formulated in isotonic 0.9% saline to pH = 7.2 and administered intravenously (i.v.) via a catheter into the tail vein.

### PET Imaging

Aliquots of the formulated [^64^Cu]PDC-**1** in isotonic 0.9% saline (8.51–9.44 MBq, 3–3.4 μg, 0.46–0.51 nmol, 100 μL, pH 7.2) were injected intravenously (i.v.) via a catheter into the tail vein of mice (*n* = 4/tumor model) anesthetized with 2–3% isoflurane in medical grade oxygen. Following a conscious uptake period, animals were anesthetized with 2–3% isoflurane and imaged two at a time, side by side. PET scans were acquired using an Inveon DPET scanner and CT scans using an Inveon SPECT/CT (PET: a static 15 min scan at 4 h p.i., and static 30 min scans at 24 and 48 h p.i., respectively) and analyzed using Inveon Research Workplace software. The mean weights and standard deviation (SD) of the imaging mice was 26.6 ± 1.6 g for the DX3puroβ6/DX3puro paired tumor model and 27.7 ± 3.7 g for the pancreatic BxPC-3 tumor model.

### Biodistribution

Aliquots of the formulated [^64^Cu]PDC-**1** in isotonic 0.9% saline (4.81–5.74 MBq, 1.7–2.1 μg, 0.26–0.31 nmol, 100 μL, pH 7.2) were injected i.v. as described above. Following the conscious uptake period, the mice were anesthetized (5% isoflurane), euthanized, and dissected (*n* ≥ 3/model/time point [4, 24, and 48 h]; the 48 h time point also included the imaging animals sacrificed after the PET scans). Tissues were collected, washed, weighed, and radioactivity measured in a γ-counter. Calibrated, decay-corrected radioactivity was expressed as the percentage of injected dose per gram of tissue (% ID/g). Data are reported as mean ± SD. The mean weights and standard deviation of the DX3puroβ6/DX3puro paired tumor model biodistribution mice were 24.3 ± 1.4 g at 4 h, 28.2 ± 2.9 g at 24 h, and 27.7 ± 2.6 g at 48 h. The mean weights and standard deviation of the BxPC-3 tumor model biodistribution mice were 25.1 ± 1.9 g at 4 h, 27.0 ± 1.1 g at 24 h and 27.1 ± 3.2 g at 48 h. For in vivo blocking studies, DOTA-**2** (50 mg/kg, 205 nmol, 1.4 mg in 100 μL 0.9% saline) was injected i.v. into two animals/tumor model 10 min prior to [^64^Cu]PDC-**1**. The animals were sacrificed after 4 h, tissues collected, washed, weighed, and radioactivity measured in a γ-counter. The mean weights and standard deviation for the blocking mice were 24.6 ± 0 and 27.9 ± 0.3 g for the DX3puroβ6/DX3puro paired and BxPC-3 tumor models, respectively.

### Therapy Studies

Tumor xenografts were established by subcutaneous injection of either DX3puroβ6 or DX3puro cells (3 × 10^6^ cells in 100 μL serum-free DMEM/animal) into the flank. The tumors were allowed to grow for 19 days before the start of treatment (day 0). Mice were treated with either (1) saline, (2) peptide ([^nat^Cu]**2**), 6 mg/kg, 1.12 μmol/kg), (3) non-targeted drug (MMAE, 0.3 mg/kg, 0.42 μmol/kg), or (4) PDC ([^nat^Cu]PDC-**1**, 6 mg/kg, 0.88 μmol/kg). Dosing of MMAE was 0.3 mg/kg as per maximum dose with no physiological response.^25^ The [^nat^Cu]PDC-**1** treatment groups consisted of *n* = 10/tumor model, while all other groups (saline, [^nat^Cu]**2**, and MMAE) consisted of *n* = 4/tumor model. All groups received four doses (on days 0, 3, 6, and 9) via i.v. tail vein injection of the above dose dissolved in saline (100 μL). The mean weights and standard deviation of each group was 25.9 ± 1.2 g (saline), 25.6 ± 1.5 g ([^nat^Cu]**2**), 25.8 ± 2.5 g (MMAE), 24.9 ± 1.9 g ([^nat^Cu]PDC-**1**, DX3puroβ6 tumors), and 25.7 ± 2.0 g ([^nat^Cu]PDC-**1**, DX3puro tumors) at day 0. Tumor volumes and body weights (to assess possible systemic toxicity) were measured starting on day 0, and once a week thereafter until the end of the study. Tumor volume (V) was determined according to the equation *V* = (π/6) × *L* × *W* × *H*, where *L* is the longest axis, *W* is the axis perpendicular to *L*, and *H* is perpendicular to the plane of *L* and *W*. End point determination criteria were: any axis >2 cm, active ulceration, or compromised health of the mouse (>20% loss of body weight from the start of the study). All data are represented as the mean ± SD and are plotted beginning at day 0. Survival curves were determined by Kaplan–Meier method.

### Statistical Analysis

Quantitative data are reported as mean ± SD. Statistical significance was determined with paired two-tailed Student’s *t* tests to give a significance value (*P*-value) at 95% confidence interval. A *P*-value of ≤0.05 was considered statistically significant.

## Supporting Information Available

The Supporting Information (S4–S36) provides the following information: chemical reagent list and commercial sources (S4), HPLC solvent gradient method (S4), cell culture reagent list and commercial sources (S5), general protocol for integrin α_v_β_6_ cell analysis by flow-cytometry (S6), flow-cytometry analysis of integrin α_v_β_6_ expression level in all cells (S6), immunohistochemistry staining of tumor tissue (S7), method for competitive ELISA (S7), method for cell binding and internalization assay (S8), method for in vitro cell blocking assay and data (S8–S9), molecular formula strings (SMILES, S10), HPLC chromatogram of NH_2_-**2** (S11), MALDI-TOF spectrum of NH_2_-**2** (S12), HPLC chromatogram of DOTA-**2** (S13), MALDI-TOF spectrum of DOTA-**2** (S14), HPLC chromatogram of [^nat^Cu]**2** (S15), MALDI-TOF spectrum of [^nat^Cu]**2** (S16), HPLC chromatogram of [^64^Cu]**2** by γ-detector (S17), HPLC chromatogram of EDTA challenged [^64^Cu]**2** by γ-detector and UV-detector (S18), HPLC chromatogram of EDTA challenged [^64^Cu]**2** by UV-detector (S19), HPLC chromatogram of [^64^Cu]**2** spiked with [^nat^Cu]**2** (S20), HPLC chromatogram of NH_2_-PDC-**1** (S21), MALDI-TOF spectrum of NH_2_-PDC-**1** (S22), HPLC chromatogram of DOTA-PDC-**1** (S23), MALDI-TOF spectrum of DOTA-PDC-**1** (S24), HPLC chromatogram of [^nat^Cu]PDC-**1** (S25), MALDI-TOF spectrum of [^nat^Cu]PDC-**1** (S26), HPLC chromatogram of [^64^Cu]PDC-**1** by γ-detector (S27), HPLC chromatogram of EDTA challenged [^64^Cu]PDC-**1** by γ-detector and UV-detector (S28), HPLC chromatogram of EDTA challenged [^64^Cu]PDC-**1** by UV-detector (S29), HPLC chromatogram of [^64^Cu]PDC-**1** spiked with [^nat^Cu]PDC-**1** (S30), table of biodistribution (% ID/g) for each tissue/tumor model (S31–S32), table of tumor-to-tissue uptake ratios for both tumor models (S33), table of biodistribution (% ID/g) for each tissue/tumor model for the blocking mice and graph (S34–S35), graph of therapy mice body weights (S36) (PDF)
(CSV)

## Abbreviations

ADC: antibody-drug conjugate
cit: citrulline
CPT: camptothecin
CT: computed tomography
Cys: cysteine
DIPEA: *N*,*N*-diisopropylethylamine
DMF: *N*,*N*-dimethylformamide
DMSO: dimethylsulfoxide
DOTA: 2,2′,2″,2‴-(1,4,7,10-tetraazacyclododecane-1,4,7,10-tetrayl)tetraacetic acid
Dox: doxorubicin
ELISA: enzyme-linked immunosorbent assay
FDA: Food and Drug Administration
Fmoc: fluorenylmethyloxycarbonyl
HATU: 1-[bis(dimethylamino)methylene]-1*H*-1,2,3-triazolo[4,5-*b*] pyridinium 3-oxid hexafluorophosphate
HPLC: high performance liquid chromatography
ID: injected dose
i.v.: intravenously
MALDI: matrix assisted laser desorption ionization
Mc: maleimide
MMAE: monomethyl auristatin E
MIP: maximum intensity projection
PABC: *para*-amino benzylcarbamate
PDC: peptide-drug conjugate
PEG: polyethylene glycol
PET: positron emission tomography
p.i.: post-injection
PBS: phosphate buffered saline
PXT: paclitaxel
pyr: pyridine
RP: reverse phase
RT: retention time
SD: standard deviation
SPPS: solid-phase peptide synthesis
TFA: trifluoroacetic acid
TIPS: triisopropylsilane
TOF: time of flight
Trt: triphenylmethyl
val: valine
