# Supplementary material for: Synthesis and Evaluation of a Monomethyl Auristatin E—Integrin αvβ6 Binding Peptide–Drug Conjugate for Tumor Targeted Drug Delivery
Source: J Med Chem. 2023 Jul 7;66(14):9842–52. doi: 10.1021/acs.jmedchem.3c00631 (PMC10388305; doi:10.1021/acs.jmedchem.3c00631)
Supplement: Supplementary file 1 — jm3c00631_si_001.pdf [file jm3c00631_si_001.pdf]

Supporting Information

Synthesis and Evaluation of a Monomethyl Auristatin E - Integrin $\alpha_v\beta_6$ Binding Peptide-Drug Conjugate for Tumor Targeted Drug Delivery

Ryan A. Davis[1], Tanushree Ganguly[1], Rebecca Harris[2], Sven H. Hausner[2], Luciana Kovacs[2], and Julie L. Sutcliffe[1-4*]

[1] Department of Biomedical Engineering, University of California, Davis, One Shields Ave., Davis, CA 95616
[2] Department of Internal Medicine, Division of Hematology/Oncology, University of California, Davis, 4150 V Street, Sacramento, CA 95817
[3] Center for Molecular and Genomic Imaging, University of California, Davis, 451 Health Sciences Drive, Davis, CA 95616
[4] Radiochemistry Research and Training Facility, University of California, Davis, 2921 Stockton Blvd., Suite 1760, Sacramento CA 95817, USA

*Email: jlsutcliffe@ucdavis.edu

Contents

Title	Page#
General chemical synthesis reagent list, purification, and characterization methodology	S4
**Table S1**. HPLC solvent gradient method	S4
Cell culture and reagents	S5
General protocol for integrin $\alpha_v\beta_6$ cell analysis by flow cytometry	S6
**Figure S1**. Flow cytometry analysis of integrin $\alpha_v\beta_6$ expression level of melanoma DX3puroβ6, DX3puro, pancreatic BxPC-3, and MIA PaCa-2 cells	S6
Immunohistochemistry method	S7
**Figure S2**. Immunohistochemistry staining of tumor tissue	S7
Competitive ELISA assay	S7
Cell binding & internalization assay	S8
In vitro cell blocking	S8

Figure S3. *In vitro* cell blocking assay. **A**. DX3puroβ6 **B**. BxPC-3 S9

Table S2. Molecular formula strings for compounds. S10

Analytical data for NH_2-**2** S11

Figure S4. HPLC chromatogram of NH_2-**2** S11

Figure S5. MALDI-TOF spectrum of NH_2-**2** S12

Analytical data for DOTA-**2** S13

Figure S6. HPLC chromatogram of DOTA-**2** S13

Figure S7. MALDI-TOF spectrum of DOTA-**2** S14

Analytical data for [^{nat}Cu]**2** S15

Figure S8. HPLC chromatogram of [^{nat}Cu]**2** S15

Figure S9. MALDI-TOF spectrum of [^{nat}Cu]**2** S16

Analytical data for [^{64}Cu]**2** S17

Figure S10. HPLC chromatogram of [^{64}Cu]**2** by γ-detector S17

Figure S11. HPLC chromatogram of EDTA challenged [^{64}Cu]**2** by γ-detector and UV-detector S18

Figure S12. HPLC chromatogram of EDTA challenged [^{64}Cu]**2** by UV-detector S19

Figure S13. HPLC chromatogram of [^{64}Cu]**2** spiked with [^{nat}Cu]**2** S20

Analytical data for NH_2-PDC-**1** S21

Figure S14. HPLC chromatogram of NH_2-PDC-**1** S21

Figure S15. MALDI-TOF spectrum of NH_2-PDC-**1** S22

Analytical data for DOTA-PDC-**1** S23

Figure S16. HPLC chromatogram of DOTA-PDC-**1** S23

Figure S17. MALDI-TOF spectrum of DOTA-PDC-**1** S24

Analytical data for [^{nat}Cu]PDC-**1** S25

Figure S18. HPLC chromatogram of [^{nat}Cu]PDC-**1** S25

Figure S19. MALDI-TOF spectrum of [^{nat}Cu]PDC-**1** S26

Analytical data for [^{64}Cu]PDC-**1** S27

Figure S20. HPLC chromatogram of [^{64}Cu]PDC-**1** by γ-detector S27

Figure S21. HPLC chromatogram of EDTA challenged [^{64}Cu]PDC-**1** by γ-detector and UV-detector S28

Figure S22. HPLC chromatogram of EDTA challenged [^{64}Cu]PDC-**1** by UV-detector S29

Figure S23. HPLC chromatogram of [^{64}Cu]PDC-**1** spiked with [^{nat}Cu]PDC-**1** S30

Table S3. Biodistribution data of [^{64}Cu]PDC-**1** in paired DX3puroβ6/DX3puro tumor model S31

Table S4. Biodistribution data of [^{64}Cu]PDC-**1** in BxPC-3 tumor model S32

Table S5. Tumor-to-tissue ratios for [^{64}Cu]PDC-**1** in paired DX3puroβ6/DX3puro and BxPC-3 tumor models for select tissues S33

Table S6. Blocking biodistribution data of [^{64}Cu]PDC-**1** in paired DX3puroβ6/DX3puro and BxPC-3 tumor models S34

Figure S24. Blocking biodistribution graph of [^{64}Cu]PDC-**1** in paired DX3puroβ6/DX3puro and BxPC-3 tumor models S35

Figure S25. Therapy study: body weight S36

General chemical synthesis reagent list, purification, and characterization methodology.

Amino acids *N*-terminally protected with a fluorenylmethyloxycarbonyl (Fmoc) protecting group and acid labile side chain protecting groups (trityl, Pbf, *tert*-butyl, or Boc) were purchased from Novabiochem (Boston, MA) or GL Biochem (Shanghai, China). The Fmoc-NH-PEG_{28} carboxylic acid was purchased from Polypure (Oslo, Norway) and the chelator 2,2′,2′′,2′′′-(1,4,7,10-tetraazacyclododecane-1,4,7,10-tetrayl)tetraacetic acid protected as tris-*tert*-butyl ester (DOTA-tris(*tert*-butyl ester) was purchased from Macrocyclics (Plano, TX). The coupling reagent 1-[bis(dimethylamino)methylene]-1*H*-1,2,3-triazolo[4,5-b]pyridinium 3-oxid hexafluorophosphate (HATU) was purchased from GL Biochem. Both MMAE and MMAE-linker (MMAE-PABC-Cit-Val-PEG_2-Mc) were purchased from MedChemExpress (Monmouth Junction, NJ). Anhydrous *N,N*-diisopropylethylamine (DIPEA) and piperidine were purchased from Sigma-Aldrich (St. Louis, MO) and used without additional purification. Triisopropylsilane (TIPS) was purchased from Alfa Aesar (Tewksbury, MA) and the copper sulfate ($CuSO_4$), ammonium acetate (NH_4OAc), and ethylenediaminetetraacetic acid (EDTA) were purchased from Sigma-Aldrich. Solvents *N,N*-dimethylformamide (DMF), dimethylsulfoxide (DMSO), methanol (MeOH), acetonitrile (ACN), and pyridine (pyr) were purchased from EMD (Burlington, MA) or Acros (Fair Lawn, NJ). The absolute ethanol was purchased from Koptec, DLI (King of Prussia, PA). Water was purified with a Millipore Integral 5 Milli-Q water system at 18.2 MΩ/cm resistivity through a 0.22 µm filter. The copper-64 ($[^{64}Cu]CuCl_2$) was purchased from the University of Wisconsin Medical Physics Department (WIMR Cyclotron Labs, Madison, WI). Measurement of radioactivity was carried out on a Wizard 1470 or Wizard2 2470 automatic γ-counter (Perkin-Elmer, Waltham, MA). Both mouse and human serum were purchased from Sigma-Aldrich. Matrigel matrix growth factor reduced (GFR) was purchased from Corning (Glendale, AZ). The 0.9% saline was purchased from Hospira (Lake Forest, IL). Imaging was performed on the Siemens Medical Solutions Inveon DPET scanner and Inveon SPECT/CT (Knoxville, TN) using the Siemens Inveon Research Workplace software.

HPLC solvent gradient method.

HPLC Solvent Gradient		
Time (min)	% Acetonitrile	% Water with 0.05% TFA
0	9	91
2	9	91
32	81	19

Table S1. RP-HPLC solvent gradient method; analytical HPLC flow rate at 1.5 mL/min and semi-preparative HPLC for purification ran at 3 mL/min.

Cell culture and reagents.

Cell culture reagents were purchased from Thermo Fisher Scientific (Gibco, Grand Island, NY) unless denoted: Dulbecco's Modified Eagle Media (DMEM), Roswell Park Memorial Institute (RPMI) 1640 media, horse serum, fetal bovine serum (FBS), penicillin-streptomycin-glutamine (PSG), penicillin-streptomycin, puromycin, and phosphate buffered saline (PBS). Tween 20 and sodium chloride (NaCl) were also purchased from Thermo Fisher Scientific (Waltham, MA). Bovine serum albumin (BSA) was purchased from EMD. Manganese chloride ($MnCl_2$), and Tris were purchased from Sigma-Aldrich. The wash buffer consisted of 2 mM of Tris buffer (pH = 7.6), 150 mM sodium chloride, 1 mM manganese chloride, and 0.1% tween20 (v/v) in deionized water. The DX3puroβ6 and DX3puro cell lines have been previously described[1] and were a gift from Dr. John Marshall; they were maintained in DMEM media, supplemented with 10% FBS, 1% penicillin-streptomycin-glutamine (100×), and 2 mg/mL puromycin. Pancreatic cells were purchased from American Type Culture collection (ATCC, Manassas, VA). The BxPC-3 cells were maintained in RPMI 1640 media supplemented with 10% FBS and 1% penicillin-streptomycin-glutamine (100×) and MIA PaCa-2 was maintained in DMEM media supplemented with 10% FBS, 2.5% horse serum, and 1% penicillin-streptomycin-glutamine (100×). The cells were maintained at 37°C and 5% CO_2. WST-1 reagent was purchased from Takara Bio USA, Inc. (San Jose, CA) and the caspase-3/7 assay kit, ApoTox-Glo Triplex Assay kit containing the Caspase-Glo 3/7 reagent, was purchased from Promega (Madison, WI). Staurosporine was purchased from Abcam (Boston, MA). WST-1 cell viability was analyzed at 450 nm using a Multiscan Ascent microplate reader (Thermo Fisher Scientific, Waltham, MA) and caspase-3/7 activity was analyzed by measuring luminescence using a Fluoroskan FL (Thermo Fisher Scientific) microplate reader.

[1]Hausner SH, Abbey CK, Bold RJ, Gagnon MK, Marik J, Marshall JF, et al., Targeted in vivo imaging of integrin $\alpha_v\beta_6$ with an improved radiotracer and its relevance in a pancreatic tumor model. Cancer Res. 2009; 69: 5843-5850.

General protocol for integrin $\alpha_v\beta_6$ cell analysis by flow cytometry.

For each cell line 3 × 10^5 cells were incubated with either the anti-integrin $\alpha_v\beta_6$ antibody 10D5 (MAB2077Z, Millipore Sigma, Temecula, CA) or the IgG2a-κ-isotype control (clone: MOPC-173, #400202, Biolegend, San Diego, CA) at 4˚C for 1 h, followed by incubation with the secondary antibody Alexa Fluor 488-goat-anti-mouse (10 µg/mL, Invitrogen, Waltham, MA) at 4˚C for 30 min and then washed with wash buffer (3×). The fluorescent signal from the secondary antibody was measured on a FACS Canto instrument (BD, Franklin Lakes, NJ) or on a LSRFortessa instrument (Becton Dickinson, Franklin Lakes, NJ) and the data processed using FlowJo v10.6.0 (BD).

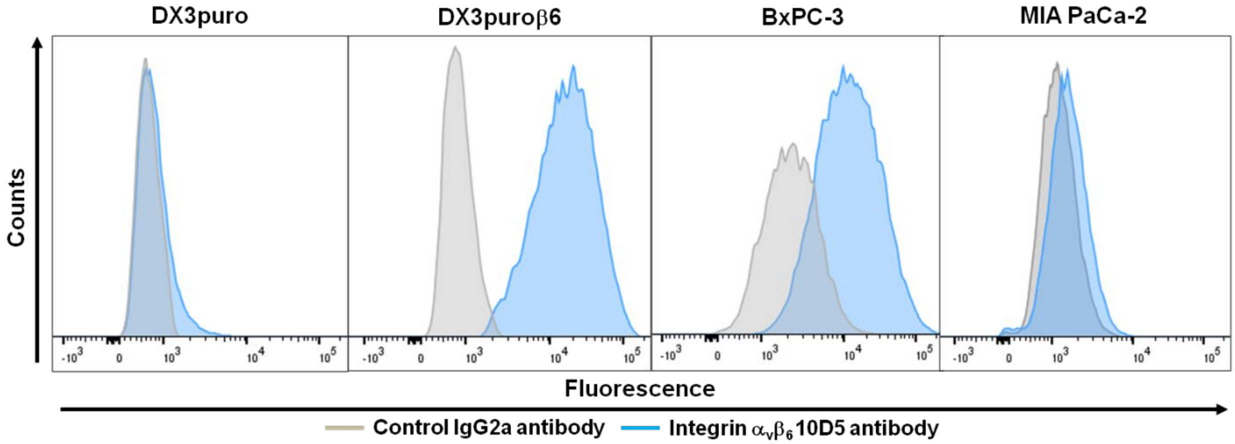

Figure S1. Flow cytometry analysis of integrin $\alpha_v\beta_6$ expression level of melanoma cells DX3puro ($\alpha_v\beta_6$ -) and DX3puroβ6 ($\alpha_v\beta_6$ +), and pancreatic cells BxPC-3 ($\alpha_v\beta_6$ +) and MIA PaCa-2 ($\alpha_v\beta_6$ -) stained with the integrin $\alpha_v\beta_6$ antibody 10D5 (blue), and control IgG2a antibody (gray).

Immunohistochemistry method

Harvested tumors were fixed in 10%-buffered formalin (Fisher Scientific) after a day and then 70% ethanol. Tissue was then frozen in a freezing medium (Tissue-Tek, Sakura Finetek; Torrence, CA) and sectioned using a Leica CM1850 cryostat (Leica Microsystems; Buffalo Grove, IL) in 20 μm slices and stained using anti-integrin β6 antibody (Calbiochem Clone 442.5C4; Burlington, MA; 1:200 dilution) on a DAKO link autostainer (Agilent; Santa Clara, CA).

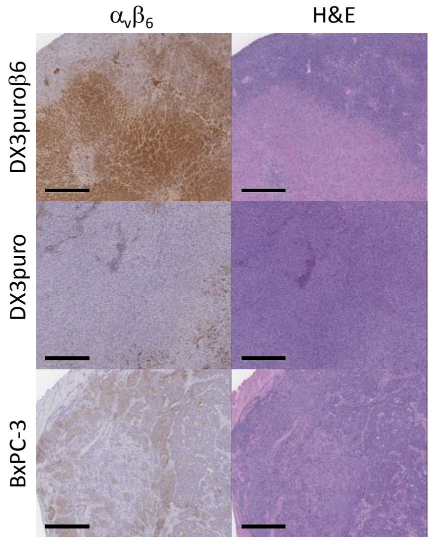

Figure S2. Immunohistochemistry staining of tumor tissue. Histology slices from tumors after immunohistochemistry staining for $\alpha_v\beta_6$ and H&E, viewed at 4× magnification. Scale bar = 500 μm.

Competitive ELISA assay.

Affinity for the integrin $\alpha_v\beta_6$ was determined by competitive binding ELISA of DOTA-PDC-**1** against biotinylated-LAP (G&P Biosciences, Santa Clara, CA). Briefly, in a 96 well Nunc Immuno maxisorp plate (Corning, Glendale, AZ), capturing anti-α_v antibody P2W7 (5 µg/mL, Abcam, Boston, MA) was plated (50 µL/well) at 37°C for 1 h, washed with PBS (3×), and blocked overnight with blocking buffer (300 µL/well, 0.5% BSA (w/v), 0.1% Tween 20, in PBS). It was then washed with wash buffer that consisted of 2 mM of Tris buffer (pH = 7.6), 150 mM sodium chloride, 1 mM manganese chloride, and 0.1% tween20 (v/v) in deionized water (3×). Purified integrin $\alpha_v\beta_6$ (R&D Systems, Minneapolis, MN) in conjugate buffer (50 µL/well, 20mM Tris, 1 mM $MnCl_2$, 150 mM NaCl, 0.1% Tween, 1% BSA in water) was then added to each well, incubated at 37°C for 1 h, followed by washing using wash buffer (3×). Serial dilutions of DOTA-PDC-**1** stock (1 mM in 10% DMSO v/v) into PBS and biotinylated natural ligand LAP were premixed in equal volumes, added to the plate in triplicate for each concentration (50 µL/well), allowed to incubate at 37˚C for 1 h, and then washed with wash buffer (3×). A 1:1000 dilution of ExtrAvidin Horseradish Peroxidase (HRP; Fisher Scientific) was added to each well (50 µL/well), incubated at 37°C for 1 h, and then washed with wash buffer (3×). The ExtrAvidin HRP was detected with TMB-One solution (50 µL/well; Promega Corp.) for 10–15 min at room temperature. The reaction was stopped by adding 1N sulfuric acid (H_2SO_4, 50 µL/well; EMD) and the absorbance was measured in a Multiscan Ascent plate reader (Thermo Fisher Scientific) at 450 nm. The IC_{50} of DOTA-PDC-**1** was determined by fitting to sigmoidal dose-response model in GraphPad Prism 5.0 (GraphPad, La Jolla, San Diego, CA). For the positive control DOTA-PDC-**1** was not added to the wells and for the negative controls either no biotinylated-LAP or no integrin $\alpha_v\beta_6$ was added.

Cell binding and internalization assay. Binding and internalization were determined as previously described[1]. In brief, bovine serum albumin (BSA, 0.5% w/v in PBS) was used to pretreat the assay tubes to prevent non-specific binding. Aliquots of [^{64}Cu]**2** or [^{64}Cu]PDC-**1** (≤1 μL, 7.4-18.5 KBq in 50 μL PBS, pH = 7.4) were added to a cell suspension (3.75×10^6 cells in 50 μL serum free medium), incubated for 1 h at 37˚C, and gently agitated every 5 minutes (*n* = 3/cell line/compound). The cells were pelleted by centrifugation at 200×g for 3 min and the supernatant collected. The cell pellet was washed with 0.5 mL serum free medium and the wash medium combined with the original supernatant. The cells were re-suspended in 0.6 mL serum free medium for γ-counting. The fraction of bound radioactivity was determined with a γ-counter (by measuring cell pellet and combined supernatants). To determine the fraction of internalized radioactivity, the cells were re-pelleted, and re-suspended in acidic wash buffer (0.2 M sodium acetate, 0.5 M sodium chloride, pH = 2.5, 300 μL, 4˚C, 5 min) to release surface bound activity, followed by a wash with PBS (300 μL). The internalized fraction was determined with a γ-counter (cell pellet *vs*. radioactivity released into supernatant). A Wizard 1470 or Wizard^2 2470 automatic γ-counter (Perkin-Elmer, Waltham, MA) was used to measure radioactivity samples.

***In vitro* cell blocking assay.**

The cell blocking assay followed the cell binding assay protocol described above with the following modification: non-radiolabeled DOTA-PDC-**1** was serially diluted from 640 nM to 10 nM in PBS. Each concentration of DOTA-PDC-**1** (25 μL) was mixed with [^{64}Cu]PDC-**1** (25 μL, 7.4-18.5 KBq in PBS) and incubated with either DX3puroβ6 or BxPC-3 cells (*n* = 3/cell line/DOTA-PDC-**1** concentration) for 1 h at 37˚C. The cells were pelleted, the supernatant removed, the cells washed, and the amount of bound radioactivity determined using the γ-counter. The fraction of bound radioactivity was calculated as radioactivity of the cell pellet divided by total radioactivity in the sample (pellet + combined supernatant). The binding values at each concentration were normalized to no binding (determined with DX3puro cells in the absence of DOTA-PDC-**1**, n = 3) set to 0%, and the maximum binding (determined with DX3puroβ6 or BxPC-3 cells, respectively, in the absence of DOTA-PDC-**1**, n = 3) set to 100%; data are expressed as the mean of normalized percentage binding ± standard deviation (SD).

[1]Hausner SH, Abbey CK, Bold RJ, Gagnon MK, Marik J, Marshall JF, et al., Targeted in vivo imaging of integrin $\alpha_v\beta_6$ with an improved radiotracer and its relevance in a pancreatic tumor model. Cancer Res. 2009; 69: 5843-5850.

***In vitro* cell binding blocking assay. A. DX3puroβ6 B. BxPC-3.**

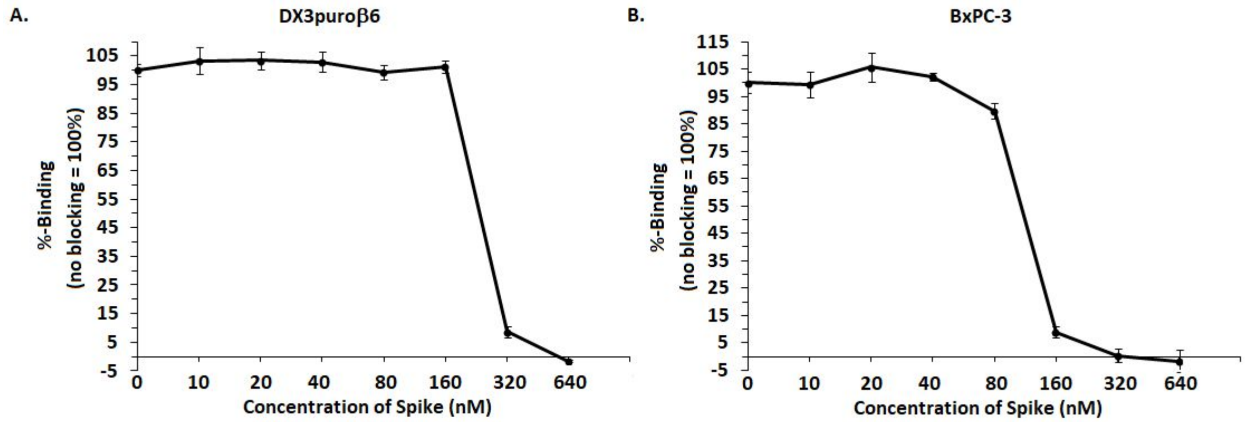

Figure S3. *In vitro* cell binding blocking assay. **A**. DX3puroβ6 **B**. BxPC-3. The data are expressed as the percentage of binding of [^{64}Cu]PDC-**1** in the presence of DOTA-PDC-**1** spike (no blocking = 0 nM, DOTA-PDC-**1** = 100% binding, n = 3 samples/cell line/spike concentration of DOTA-PDC-**1**). Data are expressed as mean $\pm$ SD. The DX3puroβ6 cells required higher concentration of non-radioactive DOTA-PDC-**1** (160-320 nM) compared to BxPC-3 cells (with lower integrin $\alpha_v\beta_6$ expression; 80-160 nM).

Molecular Formula Strings

Compound	SMILES
NH$_2$-**2**	O=C(NCCOCCOCCOCCOCCOCCOCCOCCOCCOCCOCCOCCOCCOCCOCCOCCOCCOCCOCCOCCOCCOCCOCCOCCOCCOCCOCCOCCOCCOCCOCCOCCOCCOCCOCCOCCOCCOCCOCCOCCOCCOCCOCCOCCOCCCC(N)=O)[C@H]([C@H](O)C)NC([C@@H](NC([C@@H](NC([C@@H](NC([C@@H](NC([C@@H](NC([C@H](C)NC([C@@H](NC([C@@H](NC([C@@H](NC([C@@H](NC([C@@H](NC(CNC([C@@H](NC([C@@H](NC([C@@H](NC([C@H]1CCCN1C([C@@H](NC([C@@H](NC([C@@H](NC(CCOCCOCCOCCOCCOCCOCCOCCOCCOCCOCCOCCOCCOCCOCCOCCOCCOCCOCCOCCOCCOCCOCCOCCOCCOCCOCCOCCOCCNC([C@H](CS)N)=O)=O)CC(N)=O)=O)C)=O)C(C)C)=O)=O)CC(N)=O)=O)CC(C)C)=O)CCCNC(N)=N)=O)=O)CC(O)=O)=O)CC(C)C)=O)CCC(N)=O)=O)C(C)C)=O)CC(C)C)=O)=O)CCC(N)=O)=O)CCCNC(N)=N)=O)C(C)C)=O)C)=O)CCCNC(N)=N)=O
DOTA-**2**	O=C(NCCOCCOCCOCCOCCOCCOCCOCCOCCOCCOCCOCCOCCOCCOCCOCCOCCOCCOCCOCCOCCOCCOCCOCCOCCOCCOCCOCCOCCOCCOCCOCCOCCOCCOCCOCCOCCOCCOCCOCCOCCOCCOCCOCCOCCCC(N)=O)[C@H]([C@H](O)C)NC([C@@H](NC([C@@H](NC([C@@H](NC([C@@H](NC([C@@H](NC([C@H](C)NC([C@@H](NC([C@@H](NC([C@@H](NC([C@@H](NC([C@@H](NC(CNC([C@@H](NC([C@@H](NC([C@@H](NC([C@H]1CCCN1C([C@@H](NC([C@@H](NC([C@@H](NC(CCOCCOCCOCCOCCOCCOCCOCCOCCOCCOCCOCCOCCOCCOCCOCCOCCOCCOCCOCCOCCOCCOCCOCCOCCOCCOCCOCCOCCNC([C@H](CS)NC(CN2CCN(CC(O)=O)CCN(CC(O)=O)CCN(CC(O)=O)CC2)=O)=O)=O)CC(N)=O)=O)C)=O)C(C)C)=O)=O)CC(N)=O)=O)CC(C)C)=O)CCCNC(N)=N)=O)=O)CC(O)=O)=O)CC(C)C)=O)CCC(N)=O)=O)C(C)C)=O)CC(C)C)=O)=O)CCC(N)=O)=O)CCCNC(N)=N)=O)C(C)C)=O)C)=O)CCCNC(N)=N)=O
[^{nat}Cu]**2**	OCCOCCOCCC(N)=O)[C@H]([C@H](O)C)NC([C@@H](NC([C@@H](NC([C@@H](NC([C@@H](NC([C@@H](NC([C@H](C)NC([C@@H](NC([C@@H](NC([C@@H](NC([C@@H](NC([C@@H](NC(CNC([C@@H](NC([C@@H](NC([C@@H](NC([C@H]1CCCN1C([C@@H](NC([C@@H](NC([C@@H](NC(CCOCCOCCOCCOCCOCCOCCOCCOCCOCCOCCOCCOCCOCCOCCOCCOCCOCCOCCOCCOCCOCCOCCOCCOCCOCCOCCOCCOCCNC([C@H](CS)NC(CN2CCN(CC(O)=O)CCN(CC(O)=O)CCN(C/C(O)=O/[Cu])CC2)=O)=O)=O)CC(N)=O)=O)C)=O)C(C)C)=O)=O)CC(N)=O)=O)CC(C)C)=O)CCCNC(N)=N)=O)=O)CC(O)=O)=O)CC(C)C)=O)CCC(N)=O)=O)C(C)C)=O)CC(C)C)=O)=O)CCC(N)=O)=O)CCCNC(N)=N)=O)C(C)C)=O)C)=O)CCCNC(N)=N)=O
[^{64}Cu]**2**	O=C(NCCOCCOCCOCCOCCOCCOCCOCCOCCOCCOCCOCCOCCOCCOCCOCCOCCOCCOCCOCCOCCOCCOCCOCCOCCOCCOCCOCCOCCOCCOCCOCCOCCOCCOCCOCCOCCOCCOCCOCCOCCOCCOCCOCCOCCCC(N)=O)[C@H]([C@H](O)C)NC([C@@H](NC([C@@H](NC([C@@H](NC([C@@H](NC([C@@H](NC([C@H](C)NC([C@@H](NC([C@@H](NC([C@@H](NC([C@@H](NC([C@@H](NC(CNC([C@@H](NC([C@@H](NC([C@@H](NC([C@H]1CCCN1C([C@@H](NC([C@@H](NC([C@@H](NC(CCOCCOCCOCCOCCOCCOCCOCCOCCOCCOCCOCCOCCOCCOCCOCCOCCOCCOCCOCCOCCOCCOCCOCCOCCOCCOCCOCCOCCNC([C@H](CS)NC(CN2CCN(CC(O)=O)CCN(CC(O)=O)CCN(C/C(O)=O/[64Cu])CC2)=O)=O)=O)CC(N)=O)=O)C)=O)C(C)C)=O)=O)CC(N)=O)=O)CC(C)C)=O)CCCNC(N)=N)=O)=O)CC(O)=O)=O)CC(C)C)=O)CCC(N)=O)=O)C(C)C)=O)CC(C)C)=O)=O)CCC(N)=O)=O)CCCNC(N)=N)=O)C(C)C)=O)C)=O)CCCNC(N)=N)=O
NH$_2$-PDC-**1**	OCCOCCOCCC(N)=O)[C@H]([C@H](O)C)NC([C@@H](NC([C@@H](NC([C@@H](NC([C@@H](NC([C@@H](NC([C@H](C)NC([C@@H](NC([C@@H](NC([C@@H](NC([C@@H](NC([C@@H](NC(CNC([C@@H](NC([C@@H](NC([C@@H](NC([C@H]1CCCN1C([C@@H](NC([C@@H](NC([C@@H](NC(CCOCCOCCOCCOCCOCCOCCOCCOCCOCCOCCOCCOCCOCCOCCOCCOCCOCCOCCOCCOCCOCCOCCOCCOCCOCCOCCOCCOCCNC([C@H](CSC2CC(N(CCC(NCCOCCOCCC(N[C@@H](C(N[C@@H](CCCNC(N)=O)C(NC3=CC=C(COC(N(C)[C@H](C(N[C@H](C(N([C@H]([C@H](OC)CC(N4CCC[C@H]4[C@H](OC)[C@@H](C)C(N[C@H](C)[C@@H](O)C5=CC=CC=C5)=O)=O)[C@@H](C)CC)C)=O)C(C)C)=O)C(C)C)=O)C=C3)=O)=O)C(C)C)=O)=O)C2=O)=O)N)=O)=O)CC(N)=O)=O)C)=O)C(C)C)=O)=O)CC(N)=O)=O)CC(C)C)=O)CCCNC(N)=N)=O)=O)CC(O)=O)=O)CC(C)C)=O)CCC(N)=O)=O)C(C)C)=O)CC(C)C)=O)=O)CCC(N)=O)=O)CCCNC(N)=N)=O)C(C)C)=O)C)=O)CCCNC(N)=N)=O
DOTA-PDC-**1**	O=C(NCCOCCOCCOCCOCCOCCOCCOCCOCCOCCOCCOCCOCCOCCOCCOCCOCCOCCOCCOCCOCCOCCOCCOCCOCCOCCOCCOCCOCCOCCOCCOCCOCCOCCOCCOCCOCCOCCOCCOCCOCCOCCOCCOCCOCCCC(N)=O)[C@H]([C@H](O)C)NC([C@@H](NC([C@@H](NC([C@@H](NC([C@@H](NC([C@@H](NC([C@H](C)NC([C@@H](NC([C@@H](NC([C@@H](NC([C@@H](NC([C@@H](NC(CNC([C@@H](NC([C@@H](NC([C@@H](NC([C@H]1CCCN1C([C@@H](NC([C@@H](NC([C@@H](NC(CCOCCOCCOCCOCCOCCOCCOCCOCCOCCOCCOCCOCCOCCOCCOCCOCCOCCOCCOCCOCCOCCOCCOCCOCCOCCOCCOCCOCCNC([C@H](CSC2CC(N(CCC(NCCOCCOCCC(N[C@@H](C(N[C@@H](CCCNC(N)=O)C(NC3=CC=C(COC(N(C)[C@H](C(N[C@H](C(N([C@H]([C@H](OC)CC(N4CCC[C@H]4[C@H](OC)[C@@H](C)C(N[C@H](C)[C@@H](O)C5=CC=CC=C5)=O)=O)[C@@H](C)CC)C)=O)C(C)C)=O)C(C)C)=O)C=C3)=O)=O)C(C)C)=O)=O)C2=O)=O)NC(CN6CCN(CC(O)=O)CCN(CC(O)=O)CCN(CC(O)=O)CC6)=O)=O)=O)CC(N)=O)=O)C)=O)C(C)C)=O)=O)CC(N)=O)=O)CC(C)C)=O)CCCNC(N)=N)=O)=O)CC(O)=O)=O)CC(C)C)=O)CCC(N)=O)=O)C(C)C)=O)CC(C)C)=O)=O)CCC(N)=O)=O)CCCNC(N)=N)=O)C(C)C)=O)C)=O)CCCNC(N)=N)=O
[^{nat}Cu]PDC-**1**	O=C(NCCOCCOCCOCCOCCOCCOCCOCCOCCOCCOCCOCCOCCOCCOCCOCCOCCOCCOCCOCCOCCOCCOCCOCCOCCOCCOCCOCCOCCOCCOCCOCCOCCOCCOCCOCCOCCOCCOCCOCCOCCOCCOCCOCCOCCCC(N)=O)[C@H]([C@H](O)C)NC([C@@H](NC([C@@H](NC([C@@H](NC([C@@H](NC([C@@H](NC([C@H](C)NC([C@@H](NC([C@@H](NC([C@@H](NC([C@@H](NC([C@@H](NC(CNC([C@@H](NC([C@@H](NC([C@@H](NC([C@H]1CCCN1C([C@@H](NC([C@@H](NC([C@@H](NC(CCOCCOCCOCCOCCOCCOCCOCCOCCOCCOCCOCCOCCOCCOCCOCCOCCOCCOCCOCCOCCOCCOCCOCCOCCOCCOCCOCCOCCNC([C@H](CSC2CC(N(CCC(NCCOCCOCCC(N[C@@H](C(N[C@@H](CCCNC(N)=O)C(NC3=CC=C(COC(N(C)[C@H](C(N[C@H](C(N([C@H]([C@H](OC)CC(N4CCC[C@H]4[C@H](OC)[C@@H](C)C(N[C@H](C)[C@@H](O)C5=CC=CC=C5)=O)=O)[C@@H](C)CC)C)=O)C(C)C)=O)C(C)C)=O)C=C3)=O)=O)C(C)C)=O)=O)C2=O)=O)NC(CN6CCN(CC(O)=O)CCN(CC(O)=O)CCN(C/C(O)=O/[Cu])CC6)=O)=O)=O)CC(N)=O)=O)C)=O)C(C)C)=O)=O)CC(N)=O)=O)CC(C)C)=O)CCCNC(N)=N)=O)=O)CC(O)=O)=O)CC(C)C)=O)CCC(N)=O)=O)C(C)C)=O)CC(C)C)=O)=O)CCC(N)=O)=O)CCCNC(N)=N)=O)C(C)C)=O)C)=O)CCCNC(N)=N)=O
[^{64}Cu]PDC-**1**	O=C(NCCOCCOCCOCCOCCOCCOCCOCCOCCOCCOCCOCCOCCOCCOCCOCCOCCOCCOCCOCCOCCOCCOCCOCCOCCOCCOCCOCCOCCOCCOCCOCCOCCOCCOCCOCCOCCOCCOCCOCCOCCOCCOCCOCCOCCCC(N)=O)[C@H]([C@H](O)C)NC([C@@H](NC([C@@H](NC([C@@H](NC([C@@H](NC([C@@H](NC([C@H](C)NC([C@@H](NC([C@@H](NC([C@@H](NC([C@@H](NC([C@@H](NC(CNC([C@@H](NC([C@@H](NC([C@@H](NC([C@H]1CCCN1C([C@@H](NC([C@@H](NC([C@@H](NC(CCOCCOCCOCCOCCOCCOCCOCCOCCOCCOCCOCCOCCOCCOCCOCCOCCOCCOCCOCCOCCOCCOCCOCCOCCOCCOCCOCCOCCNC([C@H](CSC2CC(N(CCC(NCCOCCOCCC(N[C@@H](C(N[C@@H](CCCNC(N)=O)C(NC3=CC=C(COC(N(C)[C@H](C(N[C@H](C(N([C@H]([C@H](OC)CC(N4CCC[C@H]4[C@H](OC)[C@@H](C)C(N[C@H](C)[C@@H](O)C5=CC=CC=C5)=O)=O)[C@@H](C)CC)C)=O)C(C)C)=O)C(C)C)=O)C=C3)=O)=O)C(C)C)=O)=O)C2=O)=O)NC(CN6CCN(CC(O)=O)CCN(CC(O)=O)CCN(C/C(O)=O/[64Cu])CC6)=O)=O)=O)CC(N)=O)=O)C)=O)C(C)C)=O)=O)CC(N)=O)=O)CC(C)C)=O)CCCNC(N)=N)=O)=O)CC(O)=O)=O)CC(C)C)=O)CCC(N)=O)=O)C(C)C)=O)CC(C)C)=O)=O)CCC(N)=O)=O)CCCNC(N)=N)=O)C(C)C)=O)C)=O)CCCNC(N)=N)=O

Table S2. Molecular formula strings for compounds.

Analytical data for NH_2-2

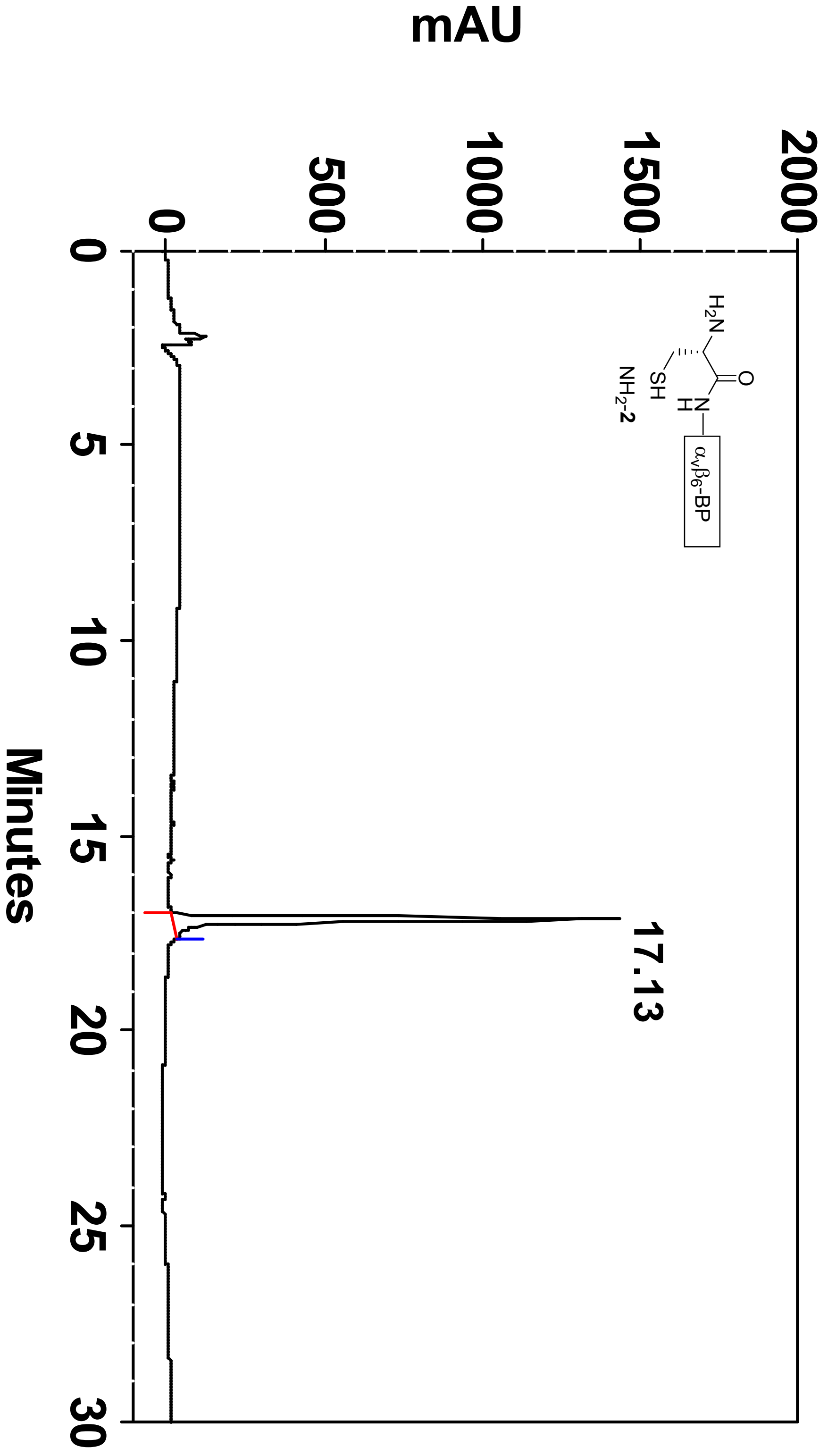

Figure S4. HPLC chromatogram of NH_2-**2** obtained on a Jupiter Proteo C_{12} column (250 mm × 4.6 mm × 4 µm) at a flow rate of 1.5 mL/min. Retention time: 17.13 min; UV: 220 nm.

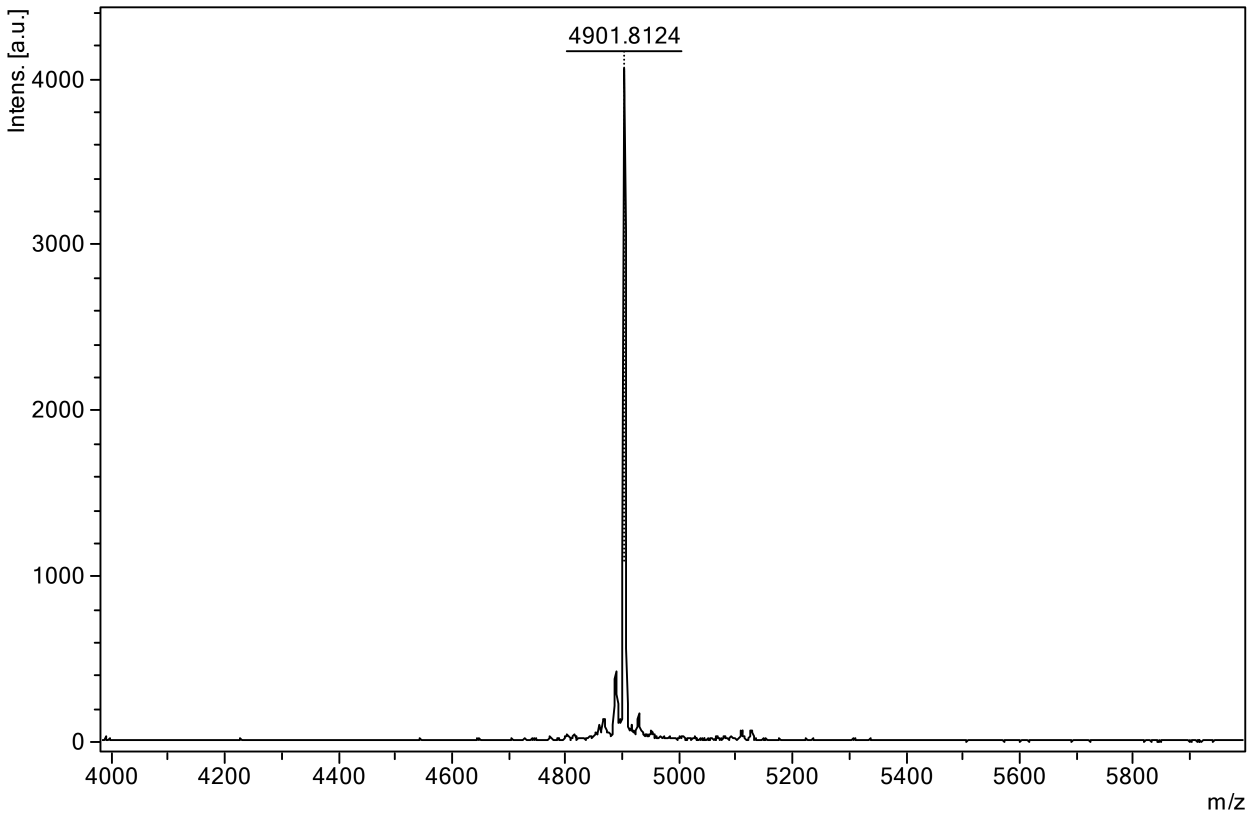

Figure S5. MALDI-TOF spectrum of NH_2-**2**.

MALDI-TOF: m/z: $[M+H]^+$ calc'd for $C_{214}H_{404}N_{37}O_{86}S$ 4901.8126; found 4901.8124.

Analytical data for DOTA-2

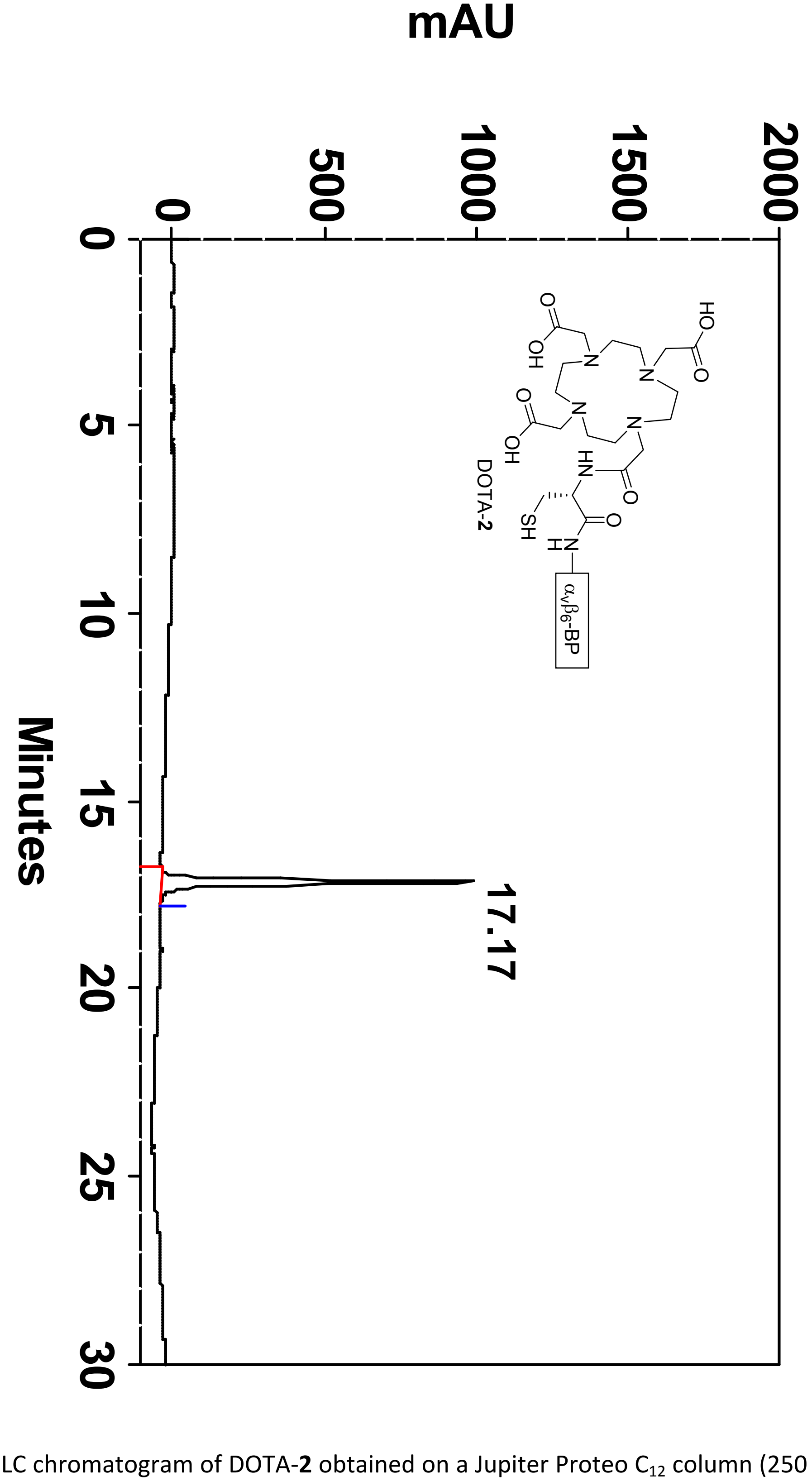

Figure S6. HPLC chromatogram of DOTA-**2** obtained on a Jupiter Proteo C_{12} column (250 mm × 4.6 mm × 4 µm) at a flow rate of 1.5 mL/min. Retention time: 17.17 min; UV: 220 nm.

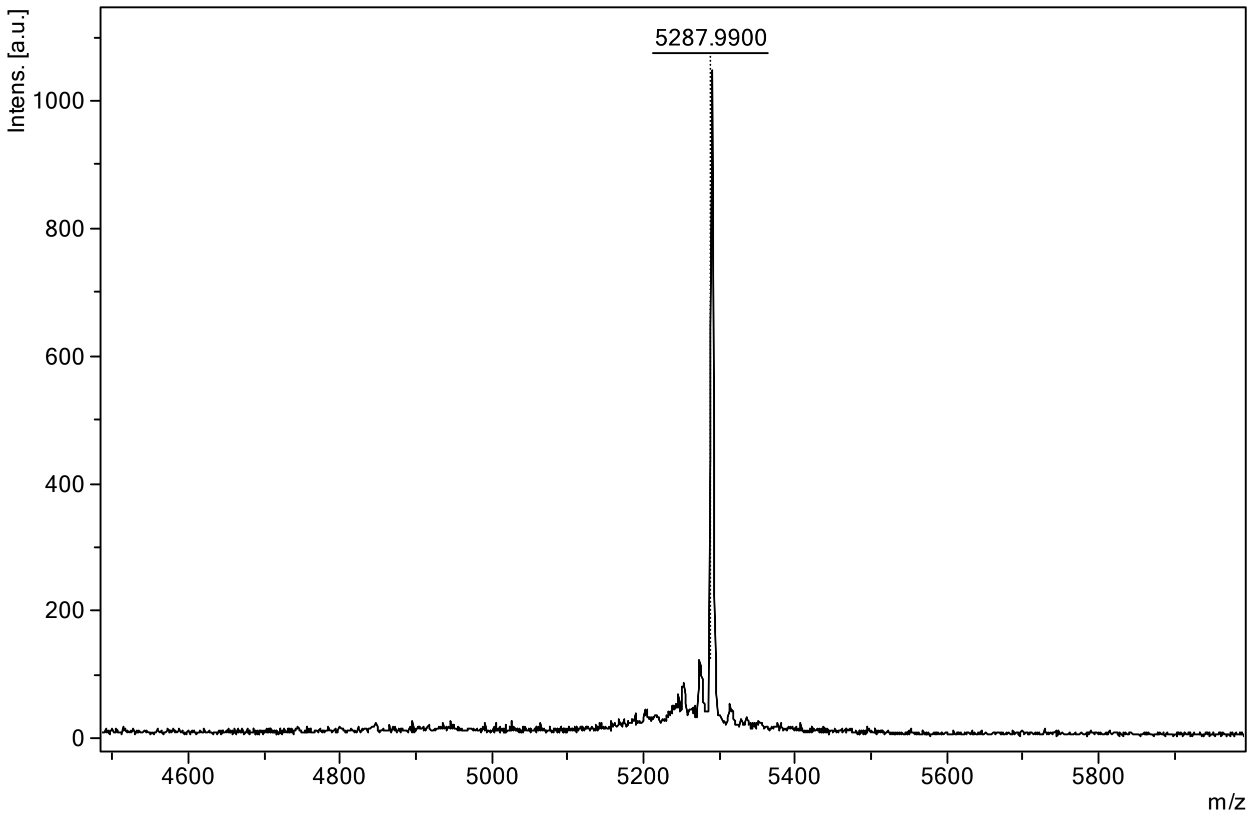

Figure S7. MALDI-TOF spectrum of DOTA-**2**.

MALDI-TOF: m/z: $[M+H]^+$calc'd for $C_{230}H_{430}N_{41}O_{93}S$ 5287.9927; found 5287.9900.

Analytical data for [^{nat}Cu]2

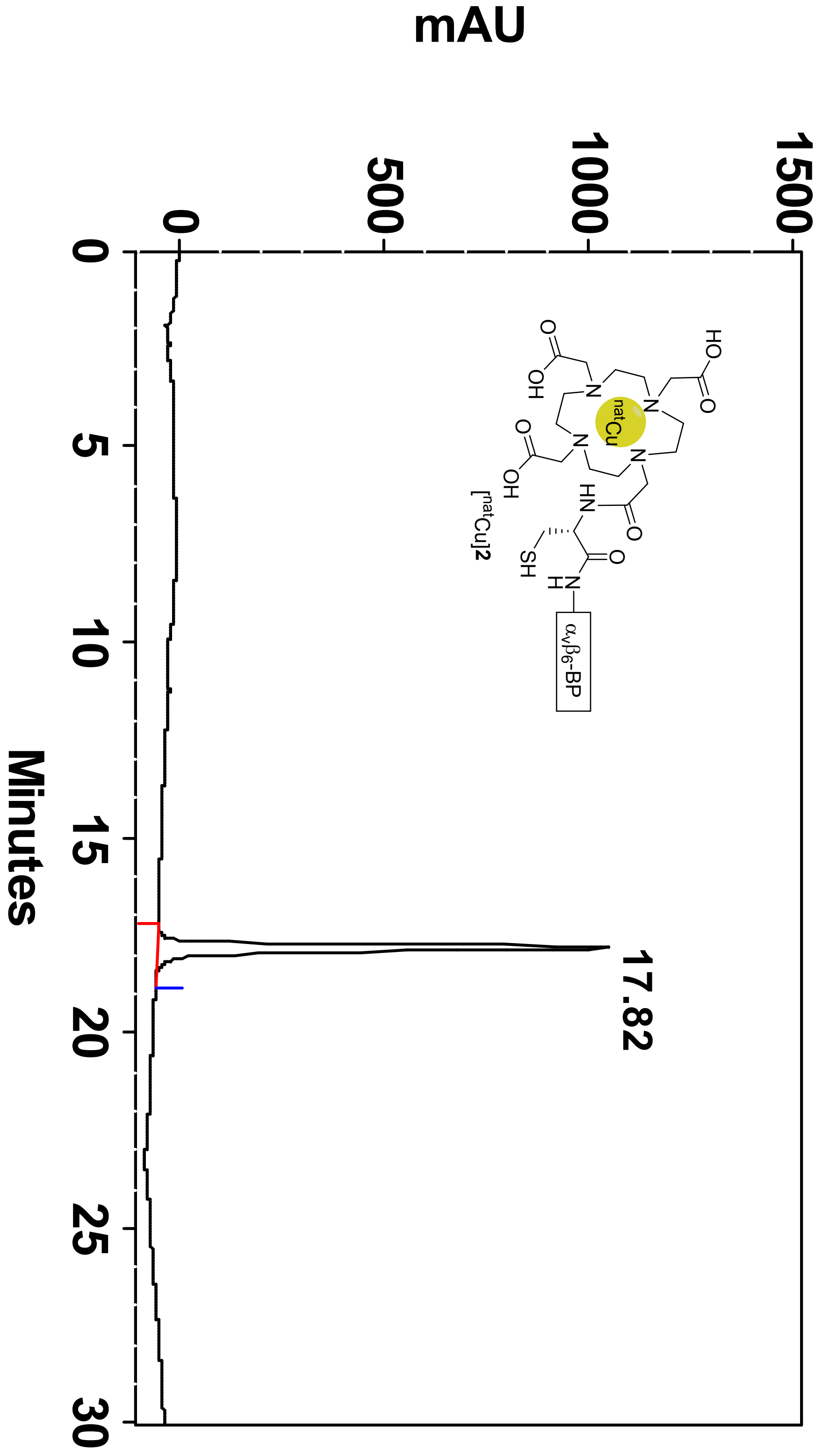

Figure S8. HPLC chromatogram of [^{nat}Cu]**2** obtained on a Jupiter Proteo C_{12} column (250 mm × 4.6 mm × 4 µm) at a flow rate of 1.5 mL/min. Retention time: 17.82 min; UV: 220 nm.

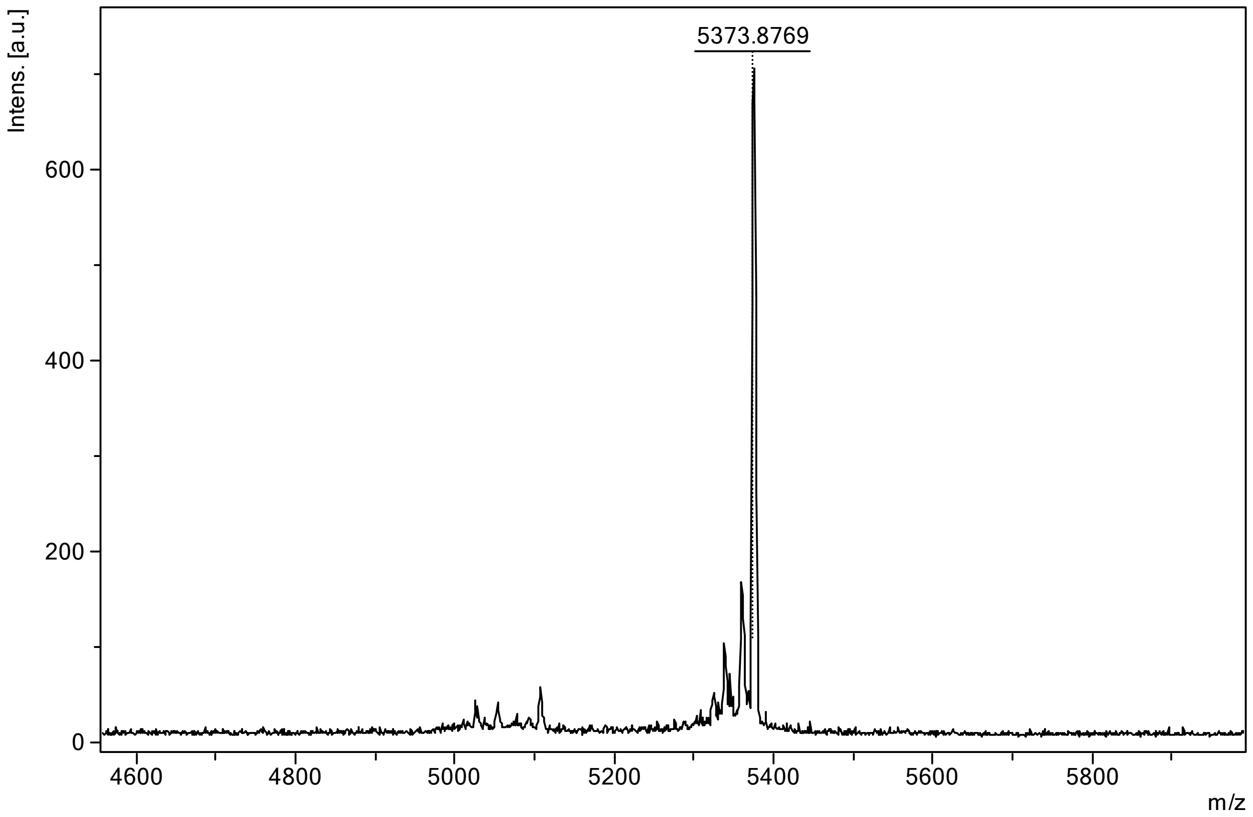

Figure S9. MALDI-TOF spectrum of [^{nat}Cu]**2**.

MALDI-TOF: m/z: $[M+Na]^+$calc'd for $C_{230}H_{429}CuN_{41}NaO_{93}S$ 5373.9076; found 5373.8769.

Analytical data for [^{64}Cu]2

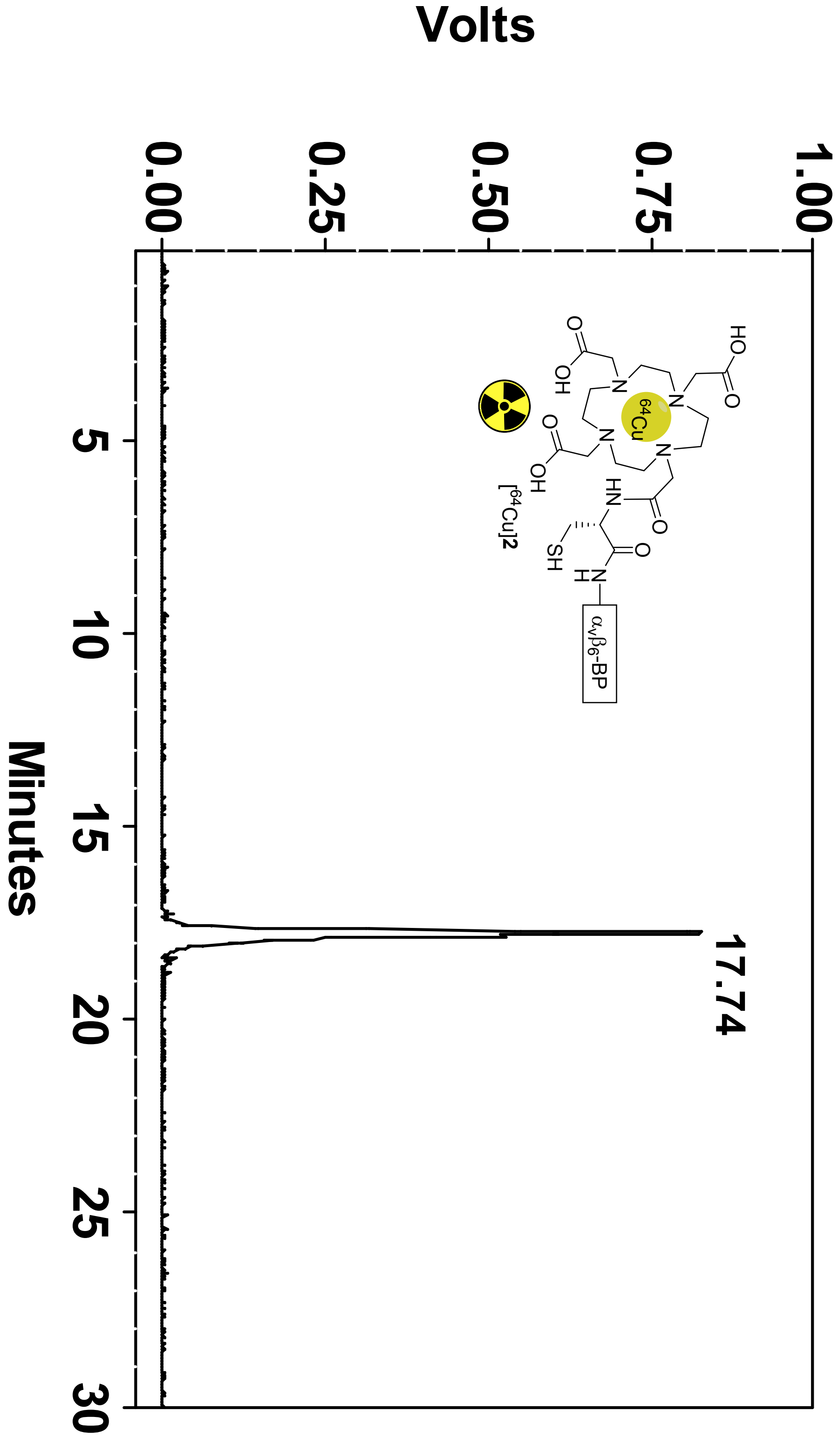

Figure S10. HPLC chromatogram of [^{64}Cu]**2** obtained on a Jupiter Proteo C_{12} column (250 mm × 4.6 mm × 4 µm) at a flow rate of 1.5 mL/min. Retention time: 17.74 min; γ-detector.

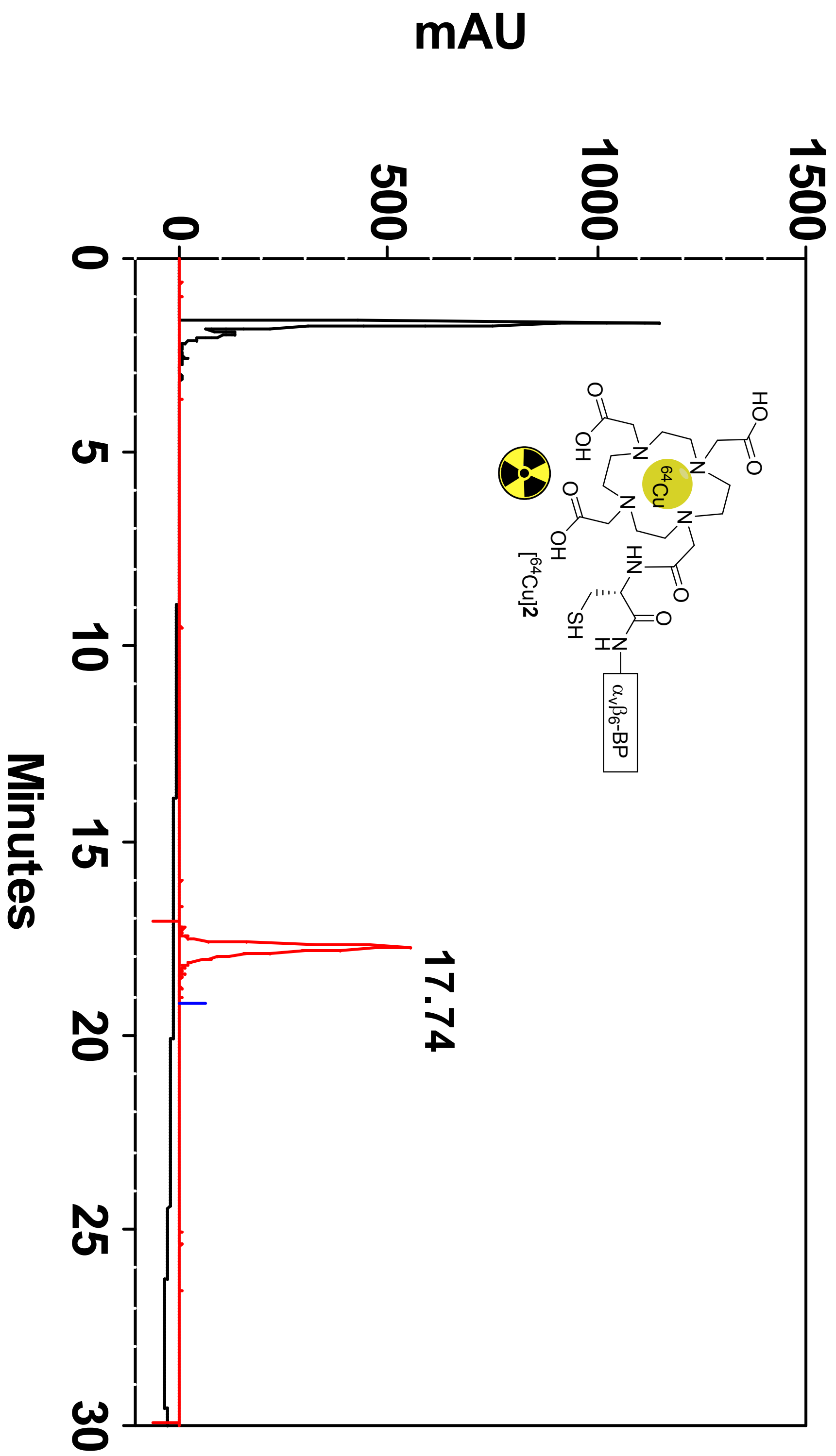

Figure S11. HPLC chromatogram of [^{64}Cu]**2** challenged with 0.1 M EDTA (50 μL, solvent front) obtained on a Jupiter Proteo C_{12} column (250 mm × 4.6 mm × 4 μm) at a flow rate of 1.5 mL/min. Retention time: 17.74 min, γ-detector; UV: 220 nm, black; γ-detector, red.

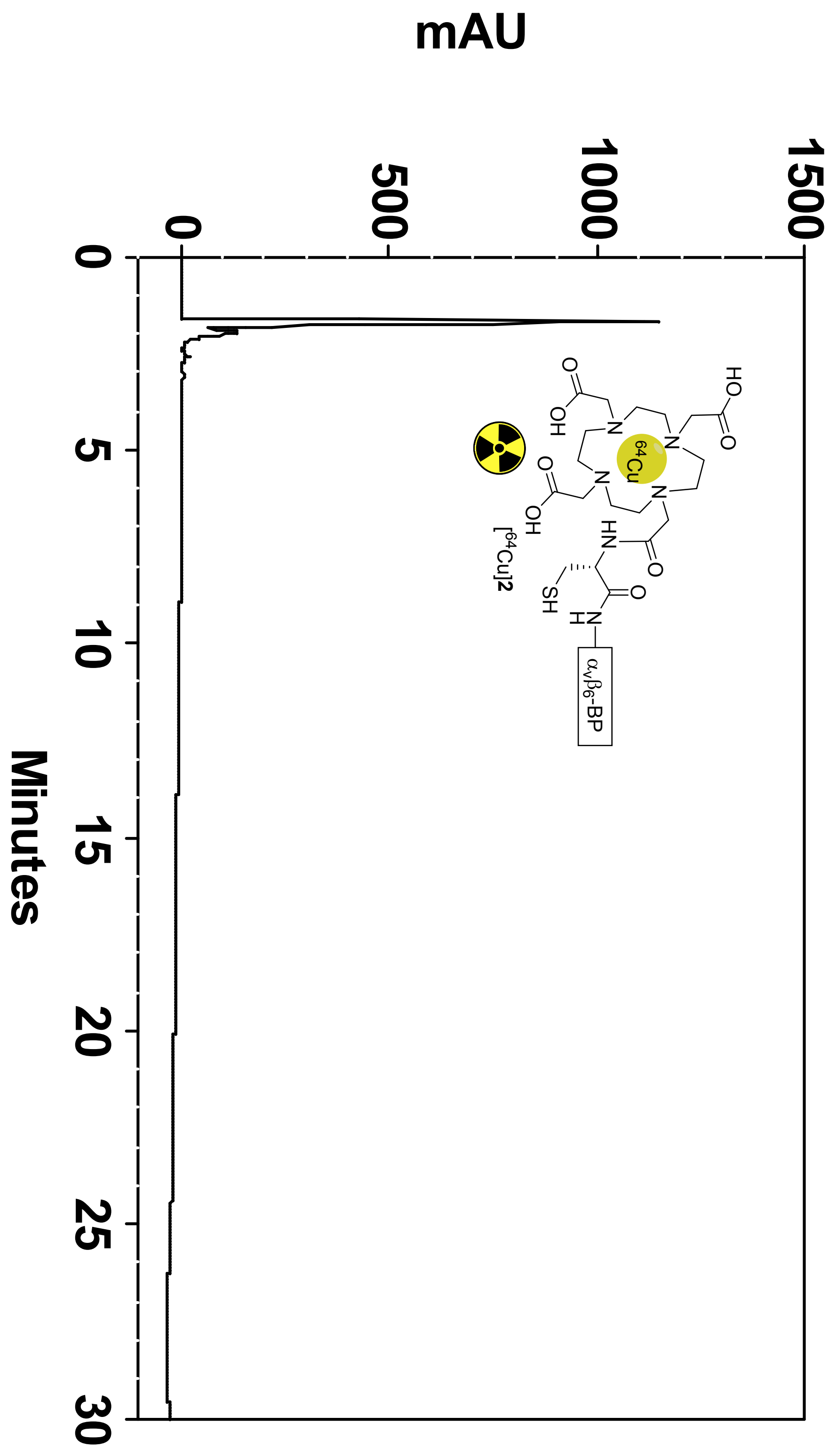

Figure S12. HPLC chromatogram of [^{64}Cu]**2** challenged with 0.1 M EDTA (50 μL, solvent front) obtained on a Jupiter Proteo C_{12} column (250 mm × 4.6 mm × 4 μm) at a flow rate of 1.5 mL/min. UV: 220 nm only.

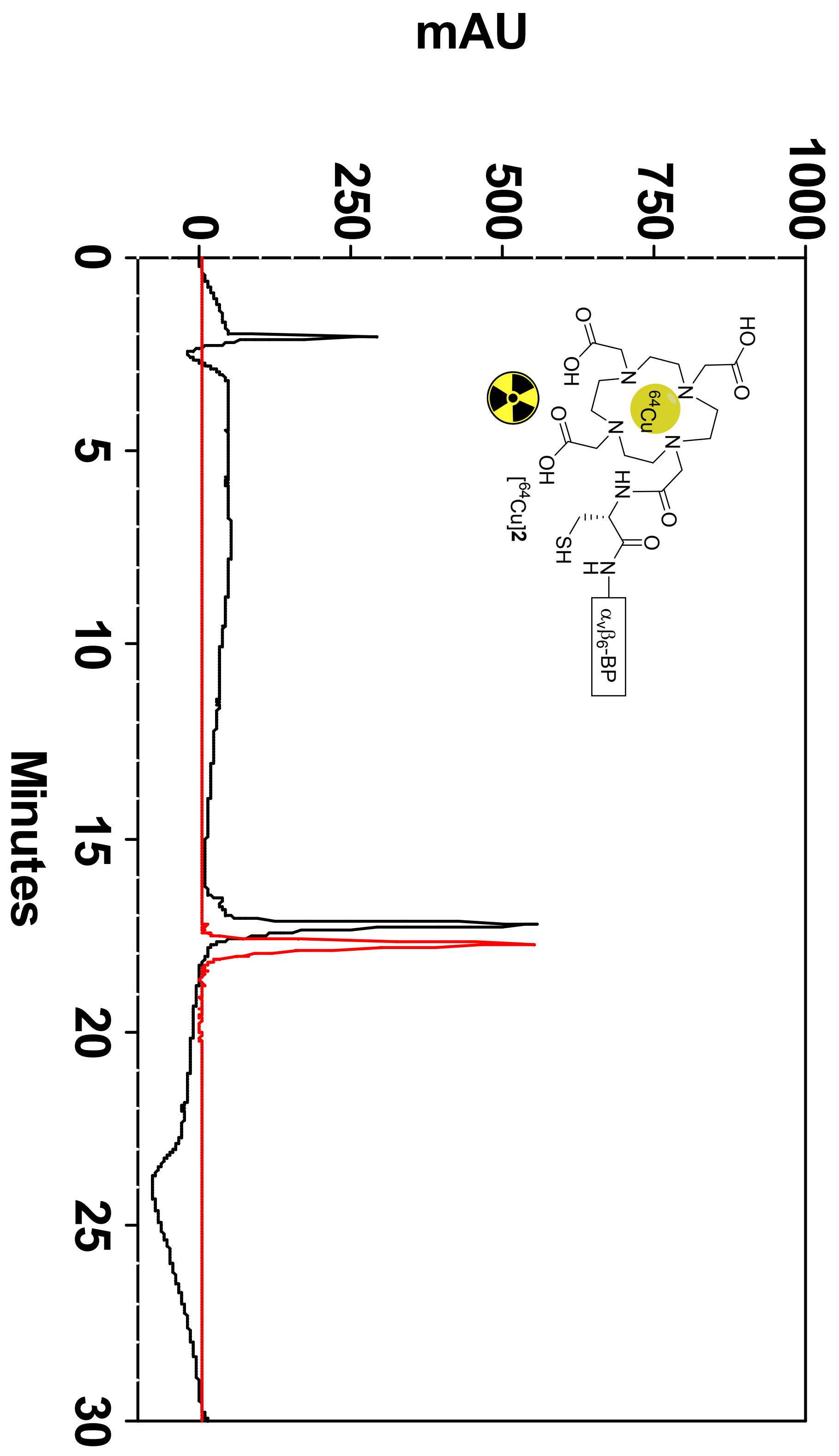

Figure S13. HPLC chromatogram of [^{64}Cu]**2** co-injected with [^{nat}Cu]**2** obtained on a Jupiter Proteo C_{12} column (250 mm × 4.6 mm × 4 µm) at a flow rate of 1.5 mL/min. UV: 220 nm, black; γ-detector, red. UV detector upstream of γ-detector.

Analytical data for NH_2-PDC-1

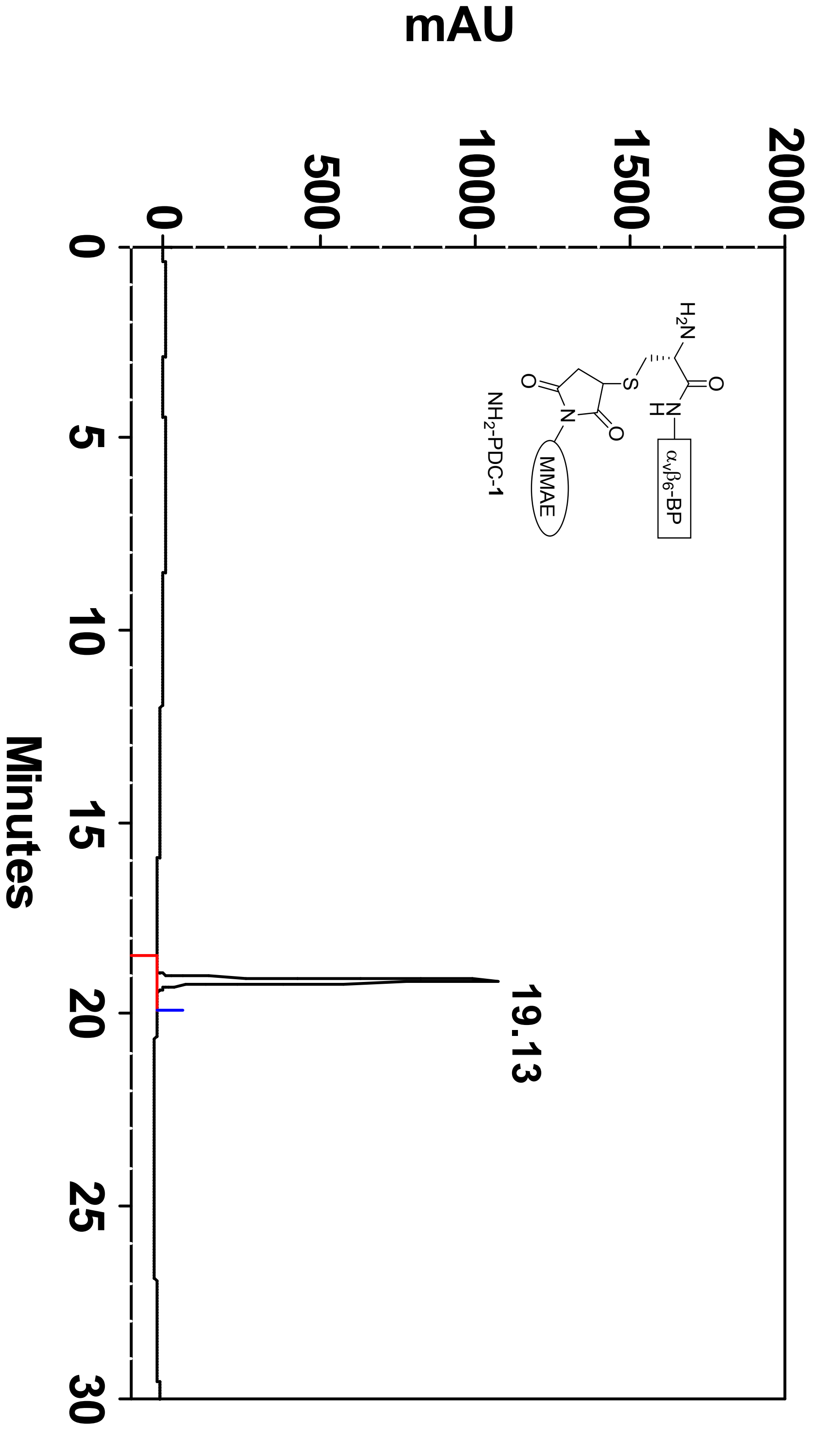

Figure S14. HPLC chromatogram of NH_2-PDC-**1** obtained on a Jupiter Proteo C_{12} column (250 mm × 4.6 mm × 4 µm) at a flow rate of 1.5 mL/min. Retention time: 19.13 min; UV: 220 nm.

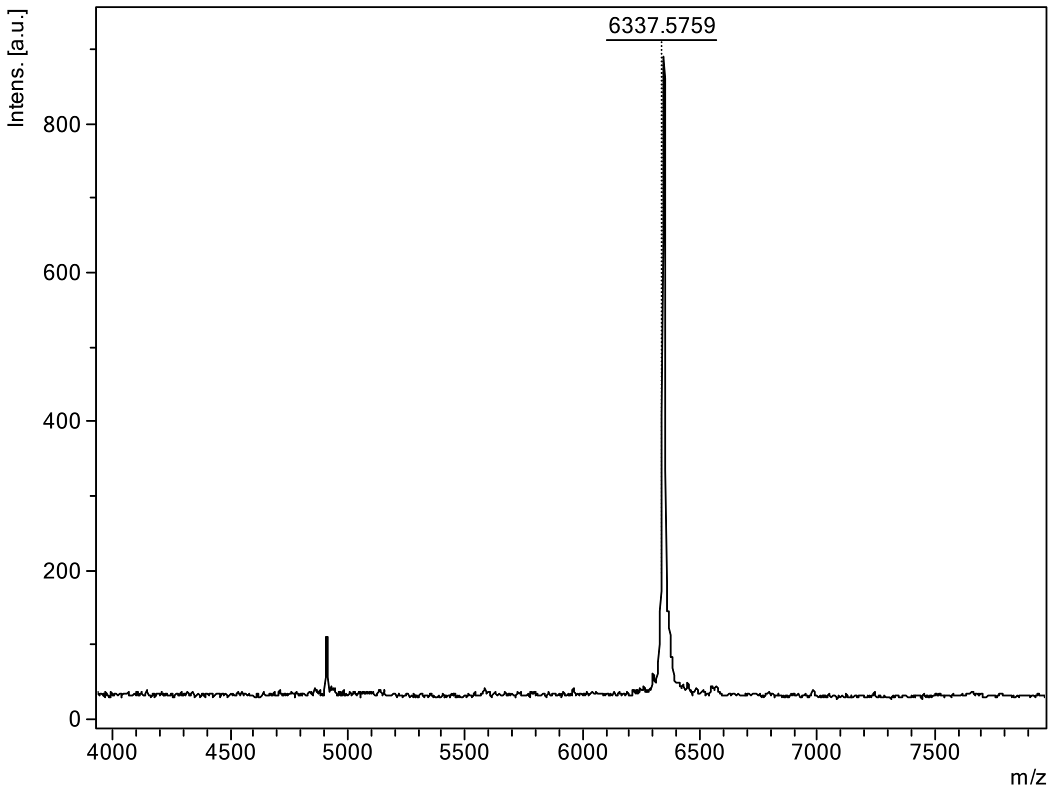

Figure S15. MALDI-TOF spectrum of NH_2-PDC-**1**.

MALDI-TOF: m/z: $[M+H]^+$ calc'd for $C_{286}H_{516}N_{49}O_{104}S$ 6337.5725; found 6337.5759.

Analytical data for DOTA-PDC-1

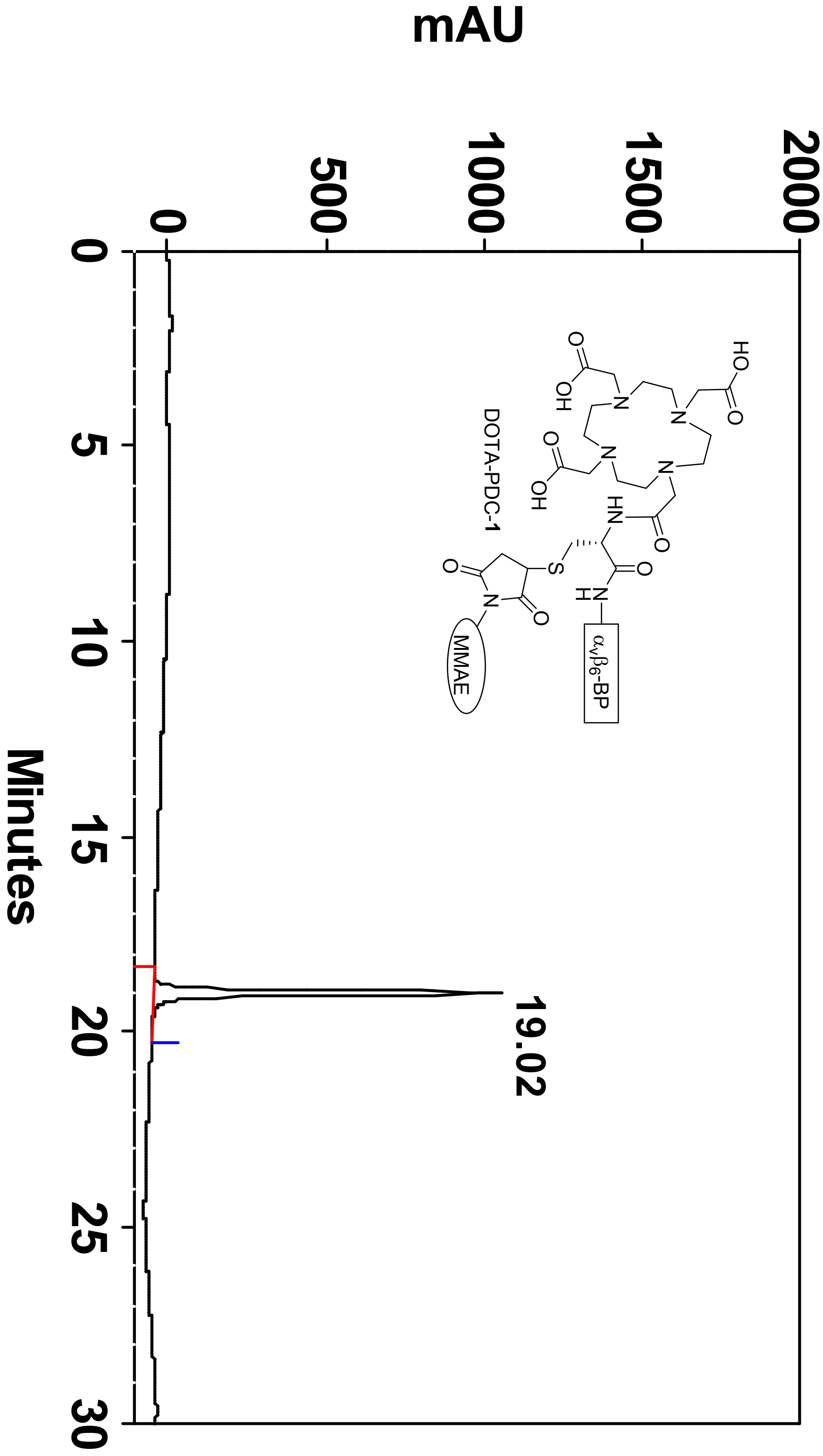

Figure S16. HPLC chromatogram of DOTA-PDC-**1** obtained on a Jupiter Proteo C_{12} column (250 mm × 4.6 mm × 4 µm) at a flow rate of 1.5 mL/min. Retention time: 19.02 min; UV: 220 nm.

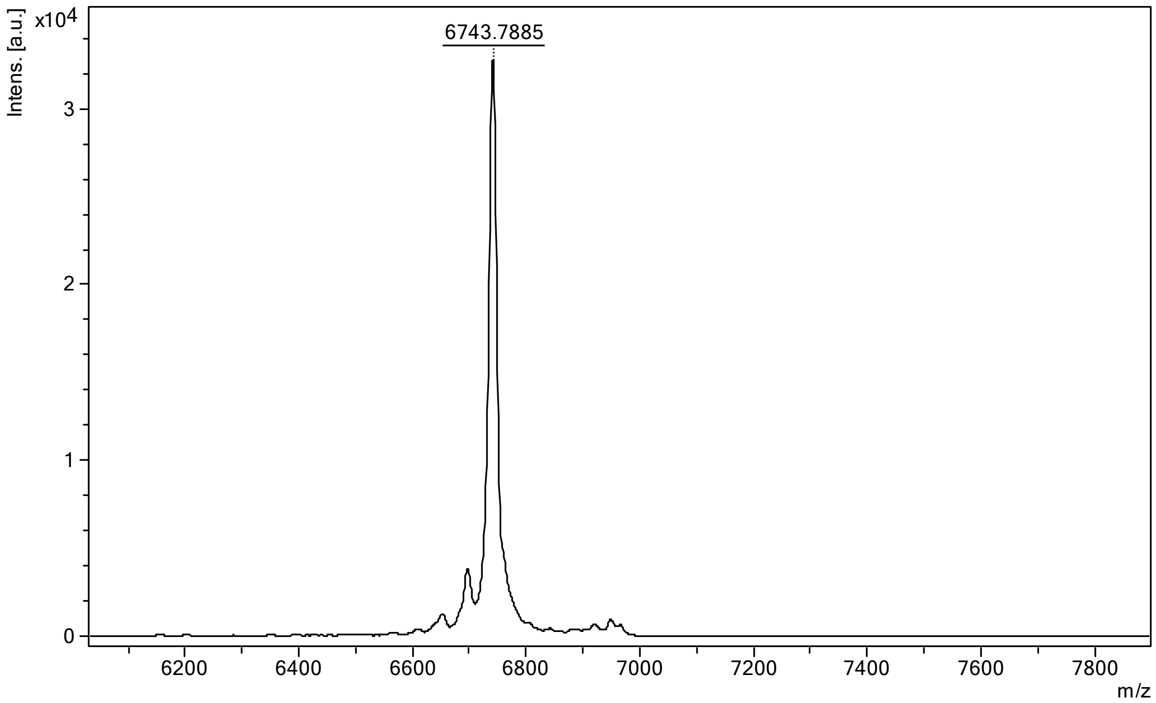

Figure S17. MALDI-TOF spectrum of DOTA-PDC-**1**.

MALDI-TOF: m/z: $[M+Na]^+$ calc'd for $C_{302}H_{541}N_{53}NaO_{111}S$ 6743.7998; found 6743.7885.

Analytical data for [^{nat}Cu]PDC-1

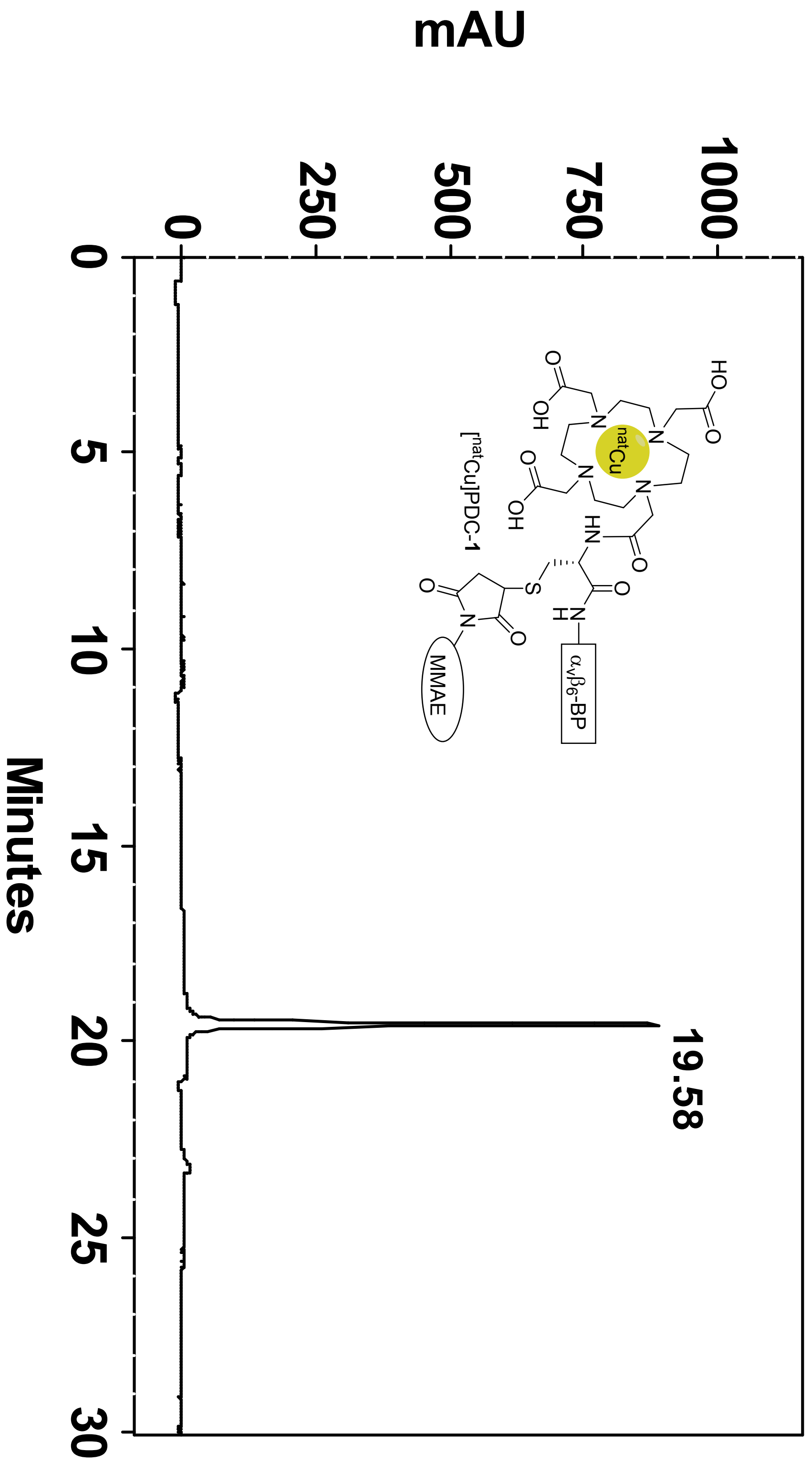

Figure S18. HPLC chromatogram of [^{nat}Cu]PDC-**1** obtained on a Jupiter Proteo C_{12} column (250 mm × 4.6 mm × 4 µm) at a flow rate of 1.5 mL/min. Retention time: 19.58 min; UV: 220 nm.

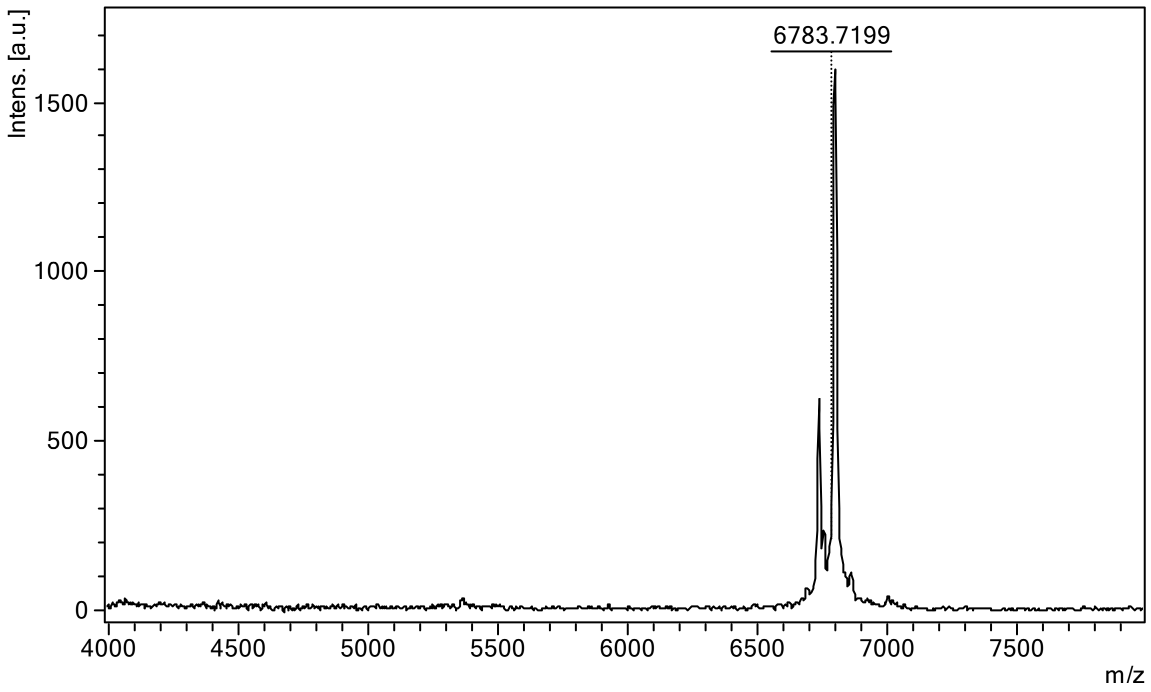

Figure S19. MALDI-TOF spectrum of [^{nat}Cu]PDC-**1**.

MALDI-TOF: m/z: $[M+H]^+$ calc'd for $C_{302}H_{541}CuN_{53}O_{111}S$ 6783.7396; found 6783.7199.

Analytical data for [^{64}Cu]PDC-1

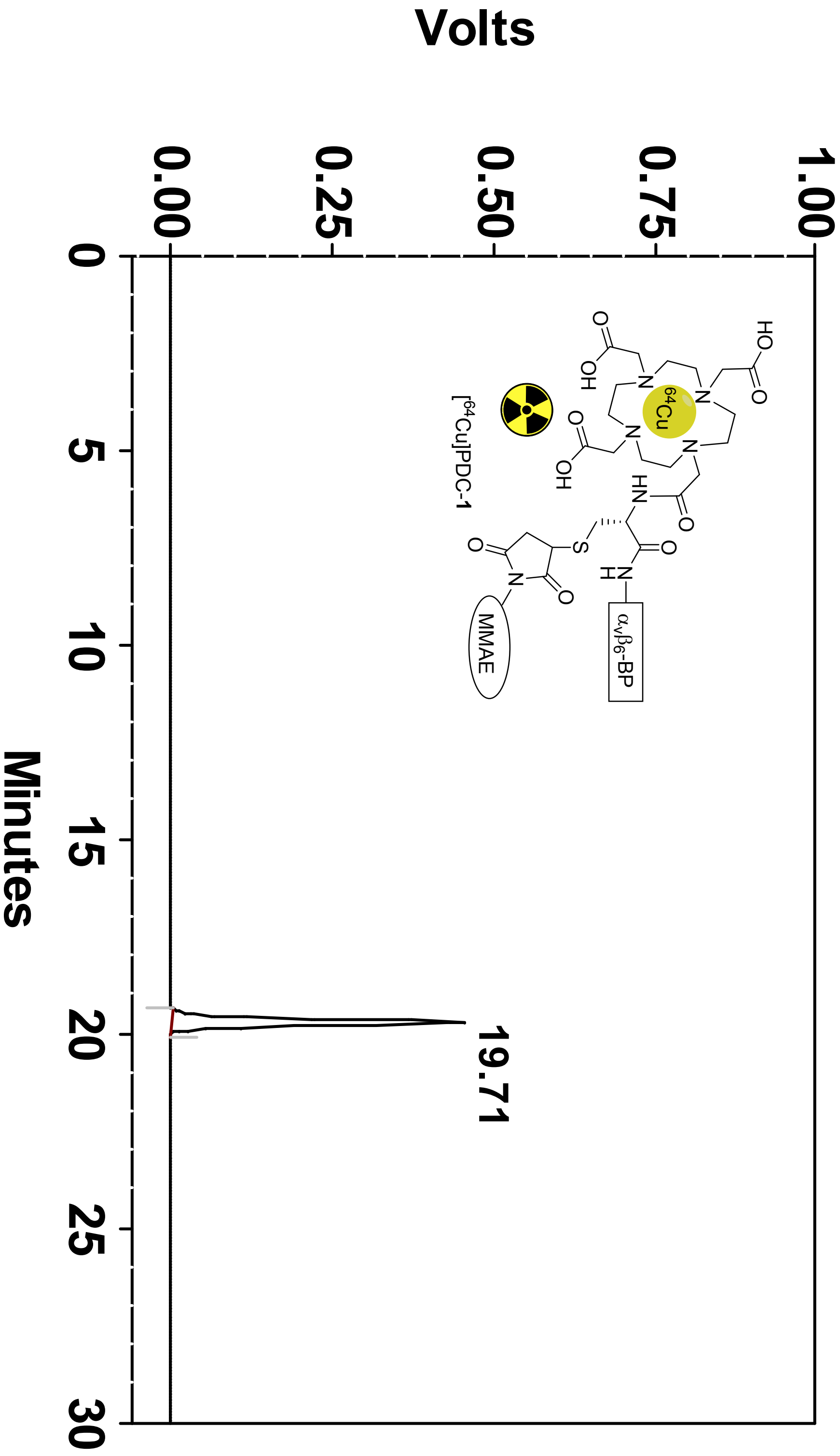

Figure S20. HPLC chromatogram of [^{64}Cu]PDC-**1** obtained on a Jupiter Proteo C_{12} column (250 mm × 4.6 mm × 4 µm) at a flow rate of 1.5 mL/min. Retention time: 19.71 min; γ-detector.

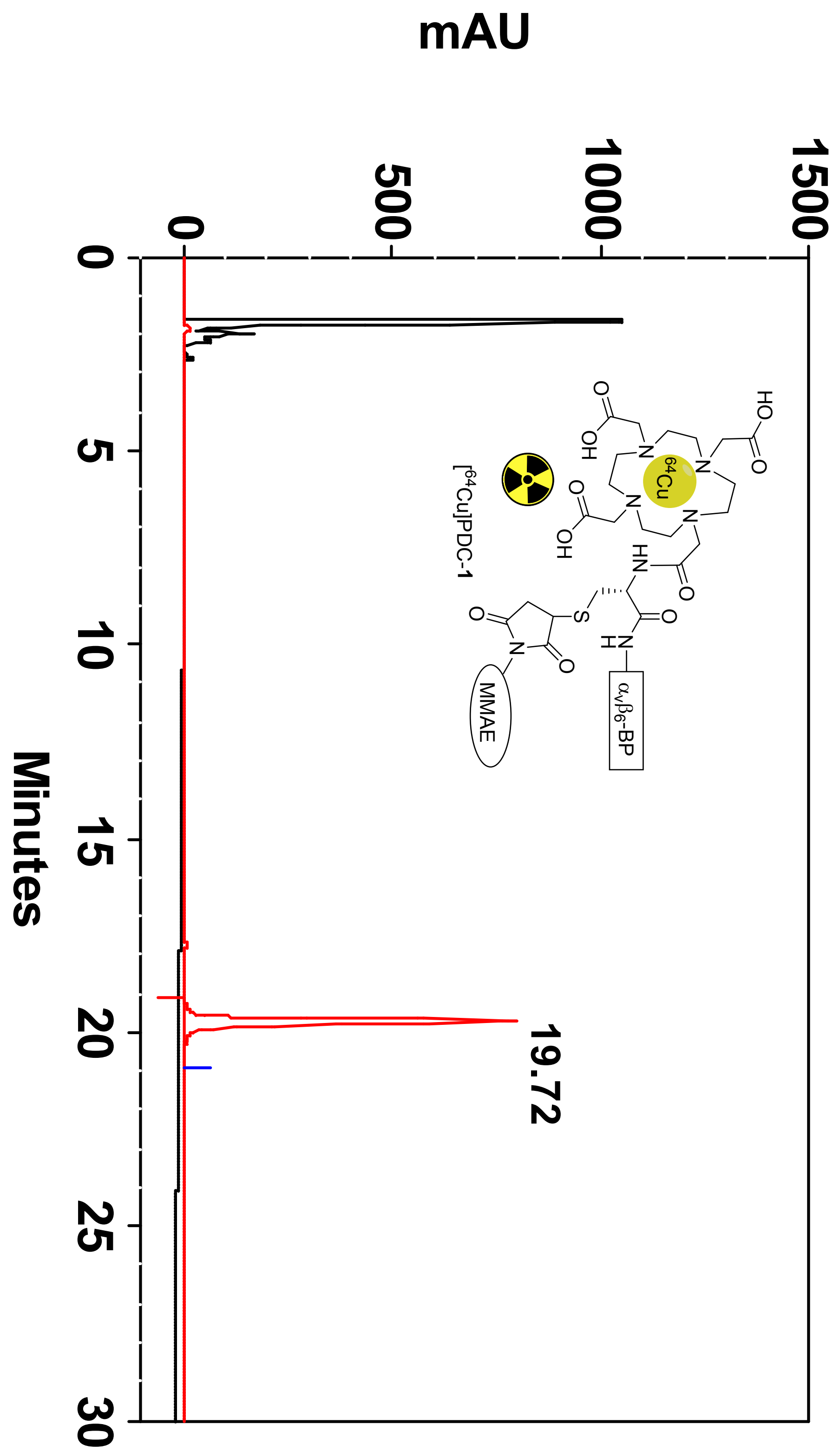

Figure S21. HPLC chromatogram of [^{64}Cu]PDC-**1** challenged with 0.1 M EDTA (50 µL, solvent front) obtained on a Jupiter Proteo C_{12} column (250 mm × 4.6 mm × 4 µm) at a flow rate of 1.5 mL/min. Retention time: 19.72 min, γ-detector; UV: 220 nm, black; γ-detector, red.

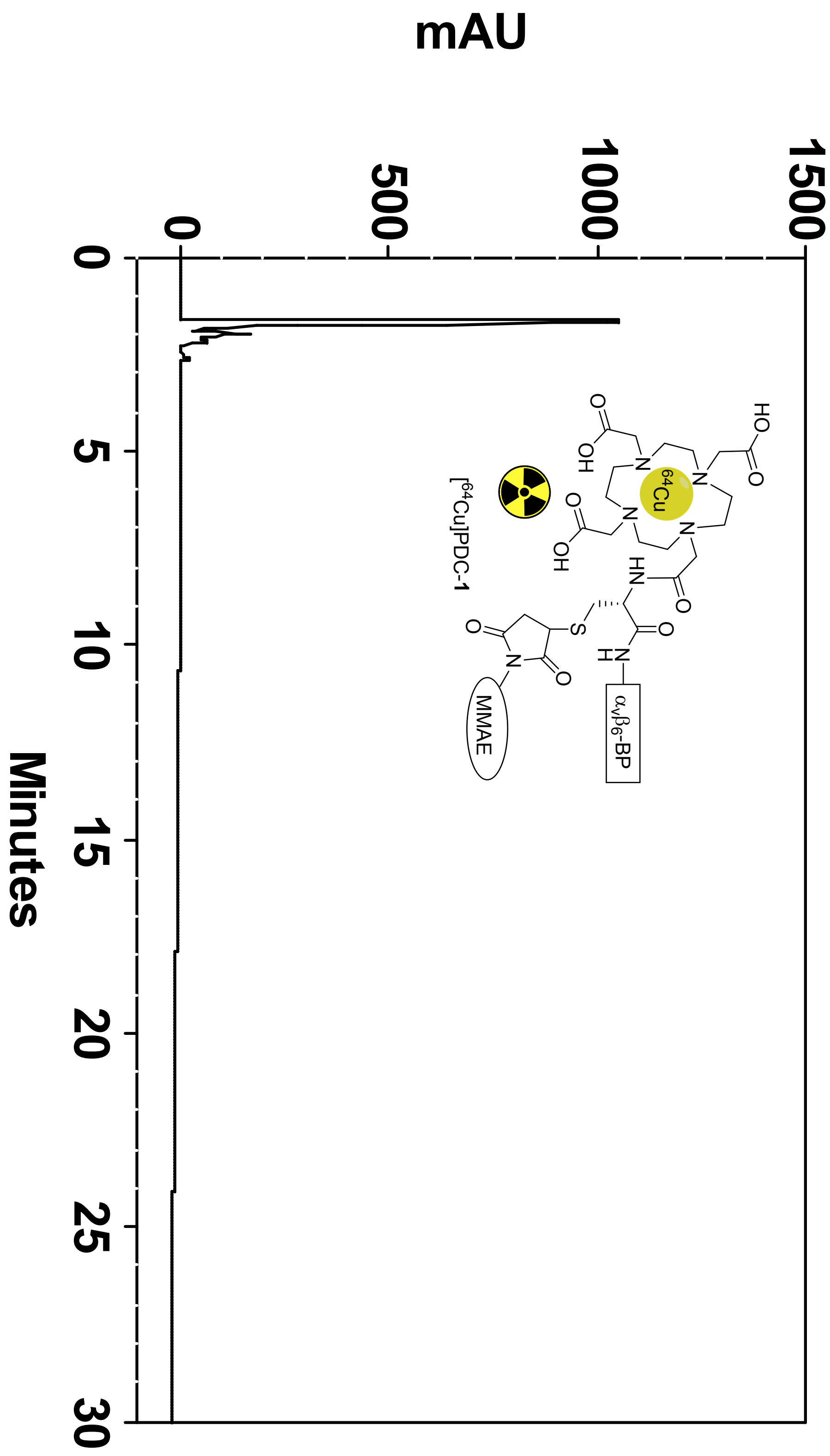

Figure S22. HPLC chromatogram of [^{64}Cu]PDC-**1** challenged with 0.1 M EDTA (50 µL, solvent front) obtained on a Jupiter Proteo C_{12} column (250 mm × 4.6 mm × 4 µm) at a flow rate of 1.5 mL/min. UV: 220 nm only.

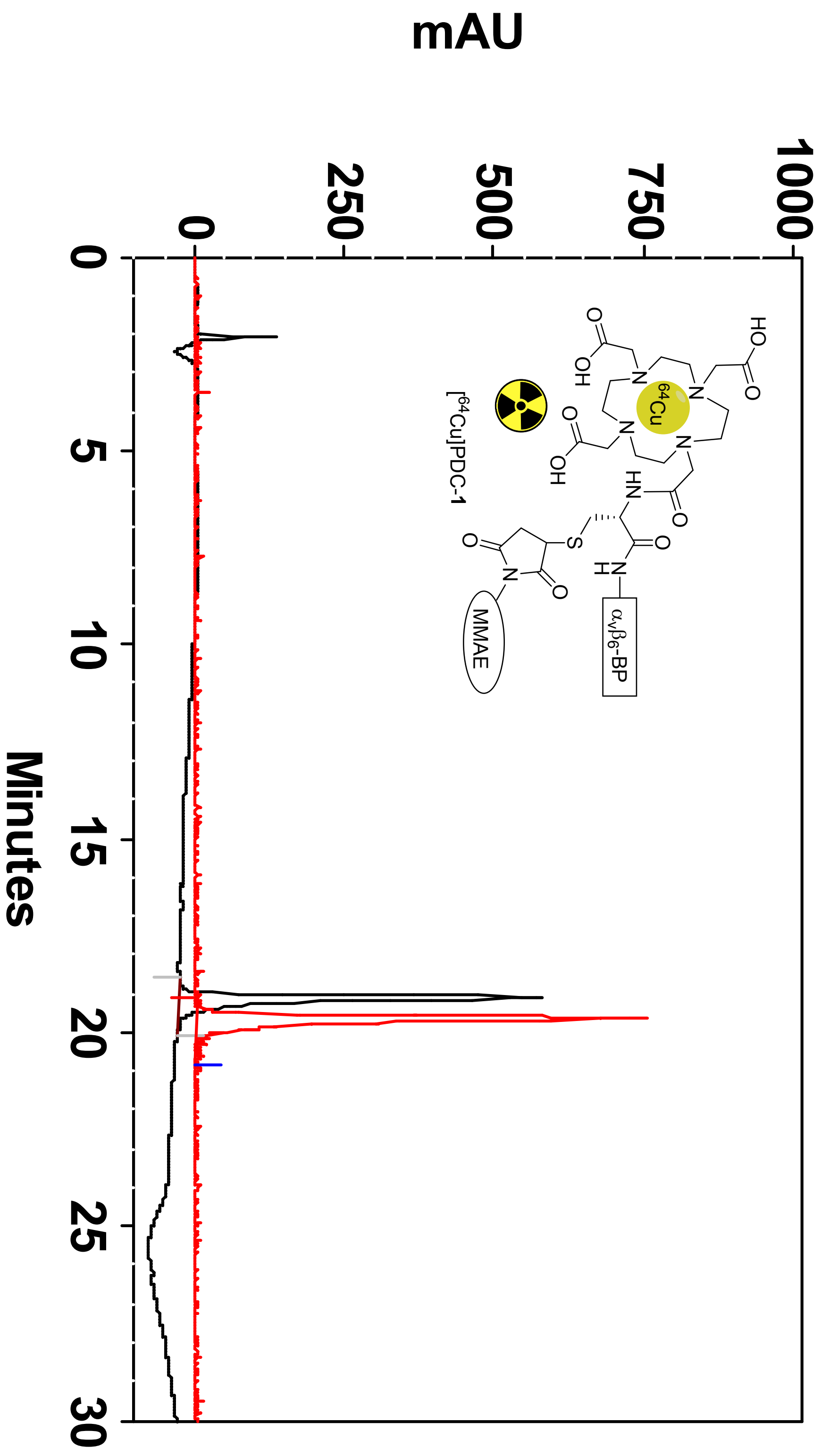

Figure S23. HPLC chromatogram of [^{64}Cu]PDC-**1** co-injected with [^{nat}Cu]PDC-**1** obtained on a Jupiter Proteo C_{12} column (250 mm × 4.6 mm × 4 µm) at a flow rate of 1.5 mL/min. UV: 220 nm, black; γ-detector, red. UV detector upstream of γ-detector.

Biodistribution data of [^{64}Cu]PDC-1 in paired DX3puroβ6/DX3puro tumor model.

Tissue	4 h	24 h	48 h
DX3puroβ6 ($\alpha_v\beta_6$ +)	4.46 ± 0.91	3.39 ± 0.56	2.53 ± 0.37
DX3puro ($\alpha_v\beta_6$ -)	0.56 ± 0.12	1.03 ± 0.12[a]	0.74 ± 0.20[b]
Blood	0.14 ± 0.02	0.29 ± 0.03	0.34 ± 0.09
Gall bladder	3.64 ± 0.79	3.16 ± 0.49	1.42 ± 0.63
Pancreas	0.36 ± 0.07	0.45 ± 0.05	0.52 ± 0.25
Liver	1.34 ± 0.29	2.25 ± 0.59	2.15 ± 0.62
Muscle	0.89 ± 0.11	0.56 ± 0.07	0.48 ± 0.14
Kidney	63.94 ± 12.48	41.22 ± 4.47	22.86 ± 5.48
Heart	0.82 ± 0.17	0.84 ± 0.15	0.85 ± 0.18
Lung	3.08 ± 0.78	2.19 ± 0.42	1.71 ± 0.44
Spleen	0.21 ± 0.08	0.58 ± 0.03	0.54 ± 0.12
Stomach	8.97 ± 0.76	4.86 ± 0.56	3.00 ± 0.56
Sm. Intestines	4.67 ± 0.66	2.73 ± 0.28	1.53 ± 0.21
Lg. Intestines	6.25 ± 0.74	3.98 ± 0.83	3.04 ± 0.73
Skin	1.96 ± 0.50	1.26 ± 0.36	1.00 ± 0.34
Bone	0.43 ± 0.18	0.38 ± 0.08	0.33 ± 0.10
Brain	0.04 ± 0.01	0.07 ± 0.01	0.09 ± 0.03
Bladder	2.12 ± 0.62	1.65 ± 0.44	1.66 ± 0.28
Urine	12.97 ± 2.40	4.57 ± 0.87	1.60 ± 0.47[c]

Table S3. Biodistribution of [^{64}Cu]PDC-**1** in mice bearing paired DX3puroβ6/DX3puro xenograft tumors. Tissue uptake is expressed as the mean of the percentage of injected dose per gram of tissue ± standard deviation (n = 4/ time point; n = 9 at 48 h); [a]n = 3, [b]n = 8, [c]n = 7.

Biodistribution data of [^{64}Cu]PDC-1 in BxPC-3 tumor model.

Tissue	4 h	24 h	48 h
BxPC-3 ($\alpha_v\beta_6$ +)	4.61 ± 1.44[a]	3.73 ± 0.44	2.93 ± 0.80
Blood	0.13 ± 0.02	0.31 ± 0.04	0.28 ± 0.04
Gall bladder	3.99 ± 1.49	2.88 ± 1.31	1.46 ± 1.09
Pancreas	0.32 ± 0.09	0.46 ± 0.04	0.47 ± 0.06
Liver	1.51 ± 0.29	1.95 ± 0.33	1.80 ± 0.30
Muscle	0.80 ± 0.09	0.68 ± 0.06	0.44 ± 0.09
Kidney	50.36 ± 3.08	41.63 ± 11.84	21.46 ± 7.01
Heart	0.79 ± 0.11	0.89 ± 0.13	0.79 ± 0.07
Lung	2.44 ± 0.30	2.30 ± 0.04	1.67 ± 0.42
Spleen	0.19 ± 0.02	0.52 ± 0.09	0.43 ± 0.08
Stomach	8.73 ± 0.66	5.94 ± 0.90	2.78 ± 0.79
Sm. Intestines	3.98 ± 0.54	2.85 ± 0.31	1.50 ± 0.23
Lg. Intestines	5.49 ± 0.47	5.21 ± 0.82	2.78 ± 1.08
Skin	1.92 ± 0.50	1.41 ± 0.25	0.79 ± 0.19
Bone	0.36 ± 0.10	0.43 ± 0.03	0.23 ± 0.11
Brain	0.04 ± 0.00	0.08 ± 0.01	0.08 ± 0.03
Bladder	2.06 ± 0.20	1.84 ± 0.45	1.28 ± 0.46
Urine	15.26 ± 1.20	4.81 ± 0.84	2.04 ± 0.97[b]

Table S4. Biodistribution of [^{64}Cu]PDC-**1** in mice bearing a BxPC-3 xenograft tumor. Tissue uptake is expressed as the mean of the percentage of injected dose per gram of tissue ± standard deviation (n = 4/ time point; n = 6 at 48 h); [a]n = 3, [b]n = 5.

Tumor-to- tissue ratios for [^{64}Cu]PDC-1 in paired DX3puroβ6/DX3puro and BxPC-3 tumor models for select tissues.

**Time point**	**4 h**		**24 h**		**48 h**	
Tumor ($\alpha_v\beta_6$ +)	DX3puroβ6	BxPC-3	DX3puroβ6	BxPC-3	DX3puroβ6	BxPC-3
Comparison Tissue						
Blood	31.9:1	35.5:1	11.7:1	12:1	7.4:1	10.5:1
Muscle	5:1	5.8:1	6.1:1	5.5:1	5.3:1	6.7:1
Liver	3.3:1	3.1:1	1.5:1	1.9:1	1.2:1	1.6:1
Kidney	0.07:1	0.09:1	0.08:1	0.09:1	0.11:1	0.14:1
Stomach	0.5:1	0.5:1	0.7:1	0.6:1	0.8:1	~1:1
Large Intestines	0.7:1	0.8:1	0.9:1	0.7:1	0.8:1	~1:1
Small Intestines	~1:1	~1:1	1.2:1	1.3:1	1.7:1	~2:1
Lung	1.5:1	~2:1	1.6:1	1.6:1	1.5:1	1.8:1
Pancreas	12.3:1	14.4:1	7.5:1	8.1:1	4.9:1	6.2:1
Tumor ($\alpha_v\beta_6$ -)	8:1	n/a	3.3:1	n/a	3.4:1	n/a

Table S5. Tumor-to-tissue ratios for [^{64}Cu]PDC-**1** for select tissues; n/a = not applicable.

Blocking biodistribution data of [^{64}Cu]PDC-1 in paired DX3puroβ6/DX3puro and BxPC-3 tumor models.

Tumor Model	DX3puroβ6/DX3puro	BxPC-3
Tissue		
Blood	0.08 ± 0.02	0.10 ± 0.02
Gall Bladder	0.28 ± 0.40	0.94 ± 0.35
Pancreas	0.08 ± 0.01	0.12 ± 0.01
Liver	1.39 ± 0.45	1.01 ± 0.16
Muscle	0.04 ± 0.00	0.07 ± 0.01
Kidney	36.43 ± 5.43	25.71 ± 2.55
Heart	0.17 ± 0.03	0.21 ± 0.06
Lung	0.39 ± 0.09	0.51 ± 0.16
Spleen	0.19 ± 0.01	0.22 ± 0.03
Stomach	0.27 ± 0.06	0.38 ± 0.04
Small Intestines	0.36 ± 0.02	0.51 ± 0.09
Large Intestines	0.51 ± 0.10	0.47 ± 0.08
Skin	0.29 ± 0.02	0.31 ± 0.02
Bone	0.10 ± 0.03	0.14 ± 0.03
Brain	0.02 ± 0.00	0.01 ± 0.01
Tumor ($\alpha_v\beta_6$ +)	0.39 ± 0.04	0.61 ± 0.05
Tumor ($\alpha_v\beta_6$ -)	0.42 ± 0.04	n/a

Table S6. Blocking biodistribution data of [^{64}Cu]PDC-**1** in paired DX3puroβ6/DX3puro and BxPC-3 tumor models. Tissue uptake is expressed as the mean of the percentage of injected dose per gram of tissue ± SD (n = 2/model, n/a = not applicable. *P* = 0.53 for DX3puroβ6 *vs*. DX3puro; *P* = 0.52 for BxPC-3 *vs*. DX3puro).

Blocking biodistribution graph of [^{64}Cu]PDC-1 in paired DX3puroβ6/DX3puro and BxPC-3 tumor models.

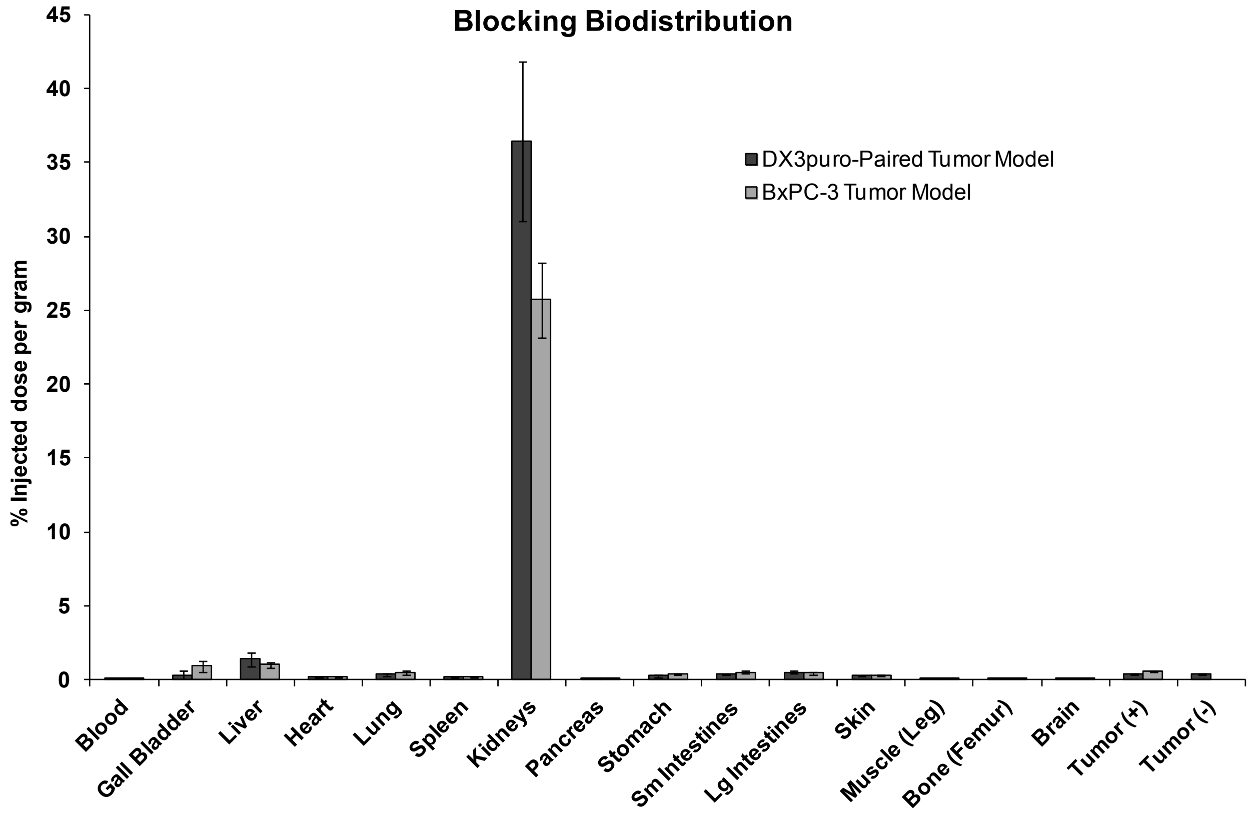

Figure S24. Graphical representation of the data in Table S22. Blocking biodistribution graph of [^{64}Cu]PDC-**1** in paired DX3puroβ6/DX3puro (■) and BxPC-3 (■) tumor models.

Therapy study: body weight.

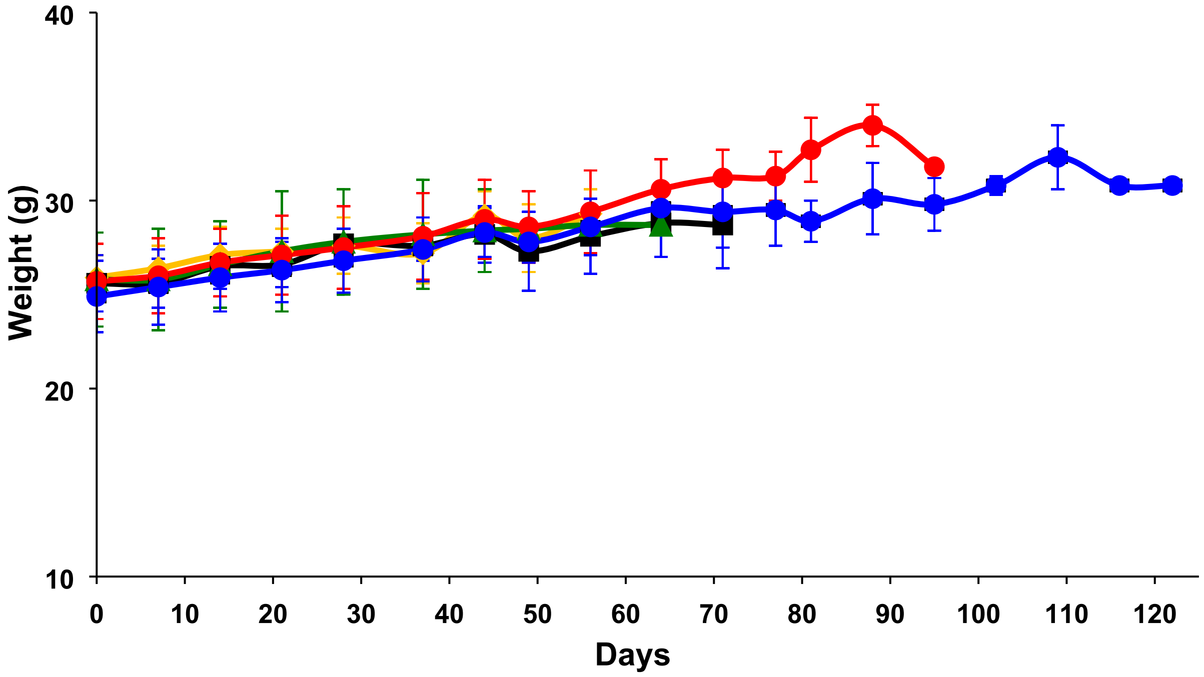

Figure S25. Body weight of mice in the therapy study. Data are displayed as mean ± SD. Control groups: saline (♦), [natCu]**2** (■), and MMAE (▲), n = 8/control group (each group consisting of half DX3puroβ6 (+), and half DX3puro (-) tumors). Treatment groups with [natCu]PDC-**1**: DX3puroβ6 (+) tumors (●) and DX3puro (-) tumors (●), n = 10/group.
